# Enhancing exosomes efficacy with engineered nanozymes for a multi-targeted combination strategy in osteoarthritis treatment

**DOI:** 10.1016/j.mtbio.2026.103118

**Published:** 2026-04-12

**Authors:** Chenyue Xu, Zhengyi Ni, Qi Wang, Handi Li, Lie Wu, Yuhang Shi, Xiaobo Chen, Ziyi Li, Huijun Kang, Yanxin Liu, Zeyu Liu, Fei Wang

**Affiliations:** a Hebei Medical University Clinical Medicine Postdoctoral Station (Hebei Medical University Third Hospital), Shijiazhuang, Hebei, 050051, China; b Department of Joint Surgery, Hebei Province Sino-US International Joint Research Center for Bone and Joint Diseases, Hebei Province Joint Degeneration and Sports Injury Technology Innovation Center, Hebei Medical University Third Hospital, Shijiazhuang, Hebei, 050051, China; c Hebei Medical University, Shijiazhuang, Hebei, 050000, China; d Center for Musculoskeletal Surgery, Charité-Universitätsmedizin Berlin, Corporate Member of Freie Universität Berlin and Humboldt-Universität zu Berlin, Berlin, 13353, Germany; e Department of Bioengineering, University of California, Los Angeles, CA, 90095, United States; f Department of Orthopaedic Surgery, Affiliated Hospital of Hebei University, Baoding, Hebei, 071000, China; g State Key Laboratory of Advanced Refractories, Wuhan University of Science & Technology, Wuhan, 430081, China

**Keywords:** Exosomes, Nanozymes, Ferroptosis, Microenvironment remodeling, Osteoarthritis

## Abstract

Osteoarthritis (OA) is a prevalent degenerative joint disease characterized by multifactorial pathological mechanisms, and remains a significant clinical challenge. Exosome therapy represents a future direction for delaying OA progression, yet its efficacy is often compromised by inflammatory microenvironment within the joints. To overcome these limitations, we present a novel combinatorial therapeutic platform that alleviates OA through a multi-targeted strategy, including the scavenging of reactive oxygen species (ROS), suppression of macrophage-driven inflammation, and inhibition of chondrocyte ferroptosis. This platform combines dental pulp stem cells-derived exosomes (Exo) with hollow mesoporous cerium oxide nanozymes, which were first loaded with curcumin and subsequently coated with hyaluronic acid, termed HA@Cur@CeO_2_. In vitro, this combination reduced intracellular ROS and promoted macrophage polarization toward the anti-inflammatory M2 phenotype, thereby remodeling the OA microenvironment and halting the inflammatory cascade. Additionally, Exo and HA@Cur@CeO_2_ nanozymes complementarily modulated ALOX12-and GPX4-dependent ferroptosis pathways in chondrocytes, with the combined approach yielding superior anti-ferroptotic effects. For in vivo assessment, Exo and HA@Cur@CeO_2_ were encapsulated within a chitosan/β-glycerophosphate hydrogel to achieve sustained release (Exo/HA@Cur@CeO_2_/Gel). This formulation significantly reduced inflammation, chondrocyte ferroptosis, cartilage degeneration, and subchondral bone remodeling, ultimately slowing OA progression. With excellent biocompatibility, this innovative combinatorial therapeutic strategy represents a comprehensive approach for enhancing Exo efficacy in OA treatment with promising translational potential.

## Graphical abstract

**Figure:**
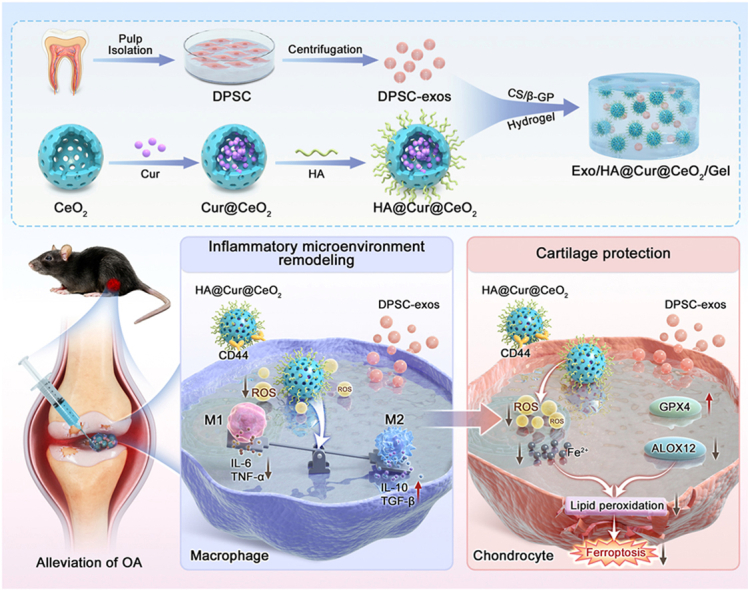

## 1 Introduction

Osteoarthritis (OA) is a prevalent degenerative joint disease characterized by progressive cartilage degradation, synovial inflammation, and subchondral bone remodeling, leading to a significant reduction in quality of life for affected individuals [1,2]. Chondrocyte ferroptosis and the inflammatory joint microenvironment are recognized as key drivers of OA pathogenesis [3,4]. However, current treatment strategies mainly focus on symptomatic relief, without targeting the underlying molecular mechanisms. This gap underscores the need for innovative disease-modifying therapies that address the root causes of OA [5]. Furthermore, given the multifactorial nature of OA, interventions aimed at a single target often yield limited efficacy. Therefore, we hypothesize that a multi-target therapeutic strategy may better address the underlying mechanisms and thereby more effectively inhibit OA progression.

Recent advances in regenerative medicine have highlighted the therapeutic potential of exosomes derived from mesenchymal stem cells (MSCs) in OA treatment. Among these, dental pulp stem cells (DPSCs)-derived exosomes (Exo) demonstrate robust anti-ferroptotic, chondrogenic, and immunomodulatory properties [[6], [7], [8]]. Furthermore, DPSCs offer advantages in terms of accessibility and safety compared to bone marrow- and adipose-derived MSCs, facilitating Exo acquisition [9]. Thus, Exo represents an ideal treatment for delaying OA progression. Nevertheless, two significant challenges persist: the therapeutic efficacy of exosomes is often compromised by inflammatory microenvironments, and their intra-articular half-life remains limited [10,11]. Therefore, investigating combinatorial therapeutic approaches holds considerable potential for advancing OA treatment.

Advances in nanotechnology and biomaterials have introduced innovative strategies for OA treatment. Notably, the integration of nanozymes, engineered nanoparticles exhibiting intrinsic enzyme-mimetic catalytic functions, into treatment modalities has shown significant promise in augmenting therapeutic outcomes and minimizing side effects [12,13]. For instance, CeO_2_ nanozymes effectively scavenge reactive oxygen species (ROS) and restore redox homeostasis within joint tissues, promoting macrophage polarization from the pro-inflammatory M1 to the anti-inflammatory M2 phenotype and attenuating inflammation [14,15]. However, the limited catalytic efficiency of CeO_2_ nanozymes under conditions of elevated oxidative stress and inflammation remains a significant barrier [16]. Hollow-structured CeO_2_ nanozymes possess a larger specific surface area and an internal cavity compared with solid CeO_2_ nanoparticles [17]. These structural features enable higher drug-loading capacity, more sustained release, and enhanced ROS-scavenging catalytic activity under OA-mimetic conditions, making them a more suitable nanozyme scaffold for OA therapy [18,19]. Curcumin (Cur), a polyphenolic compound with well-documented antioxidant and anti-inflammatory effects, is an ideal loaded drug for hollow-structured CeO_2_ [20]. Additionally, hyaluronic acid (HA), which targets the CD44 receptor overexpressed on inflammatory cells, is widely utilized as a surface ligand for inflammation-targeted nanoparticles [21]. Consequently, the development of HA-modified, Cur-loaded CeO_2_ nanozymes (HA@Cur@CeO_2_) represents a promising and rational strategy to potentiate ROS scavenging and immunomodulation.

Hydrogels are considered as reliable tissue engineering materials and in-situ drug delivery carriers [22]. The chitosan (CS)/β-glycerophosphate (β-GP) hydrogel, a natural thermosensitive matrix, is particularly attractive for OA applications due to its injectability, biocompatibility, and biodegradability [23,24]. Moreover, CS has been shown to stabilize MSC-derived exosomes and enhance their delivery in multiple diseases [25,26]. Accordingly, we integrated Exo and HA@Cur@CeO_2_ nanozyme into a CS/β-GP hydrogel (Exo/HA@Cur@CeO_2_/Gel) to prolong intra-articular retention.

**Scheme 1:**
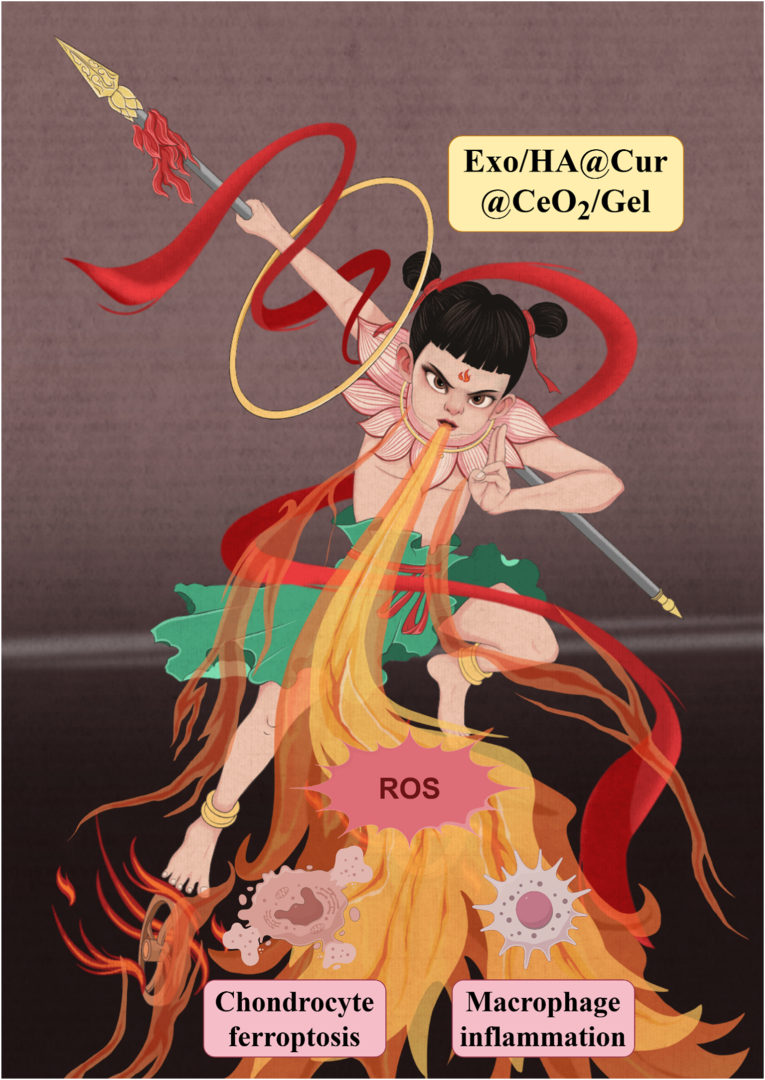
The combinatorial therapeutic platform (Exo/HA@Cur@CeO_2_/Gel) effectively attenuates OA progression by scavenging ROS, suppressing macrophage-mediated inflammation, and inhibiting chondrocyte ferroptosis.

**Fig. 1:**
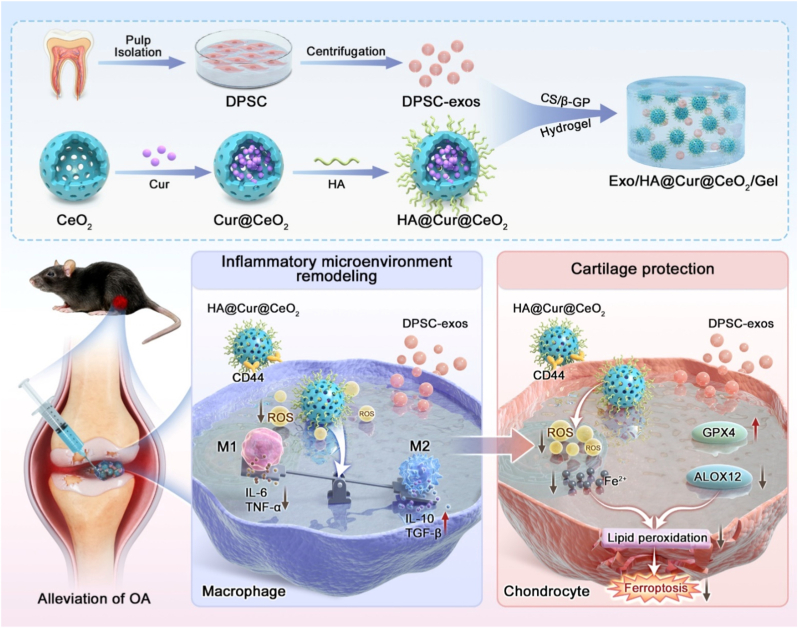
**Development of Exo/HA@Cur@CeO_2_/Gel system for the comprehensive treatment of OA**. Exo was isolated from DPSC culture supernatants, while HA@Cur@CeO_2_ nanozyme were engineered to optimize the joint microenvironment and potentiate Exo efficacy. Both Exo and HA@Cur@CeO_2_ nanozyme were then encapsulated within CS/β-GP hydrogel to facilitate sustained release. In macrophages, Exo combined with HA@Cur@CeO_2_ nanozyme cooperatively reduced ROS and promoted M2 macrophage polarization, remodeling the OA microenvironment. In chondrocytes, Exo and HA@Cur@CeO2 modulated ALOX12-and GPX4-mediated ferroptosis, with superior anti-ferroptotic effects in combination. Collectively, the Exo/HA@Cur@CeO_2_/Gel system represents a promising and comprehensive therapeutic strategy for OA.

In this study, we presented a multi-targeted combinatorial therapeutic platform designed to mitigate OA progression by scavenging ROS, suppressing macrophage-mediated inflammation, and inhibiting chondrocyte ferroptosis (Scheme 1). Specifically, we integrated Exo with an engineered HA@Cur@CeO_2_ nanozyme within an injectable hydrogel-based sustained delivery system to address key limitations of Exo-based therapy. Conceptually, our design aims to (1) remodel the inflammatory microenvironment through sustained ROS scavenging and macrophage polarization, thereby creating conditions that better preserve and potentiate Exo function, and (2) concurrently attenuate ferroptosis via complementary regulation of GPX4-and ALOX12-dependent signaling. Collectively, our strategy offering an integrated approach that simultaneously remodel inflammatory microenvironment and halt ferroptosis-driven cartilage degeneration, presenting an efficient and comprehensive strategy for OA treatment (Fig. 1).

## 2 Results and discussion

### 2.1 Characterization of Exo and nanozymes

**Fig. 2:**
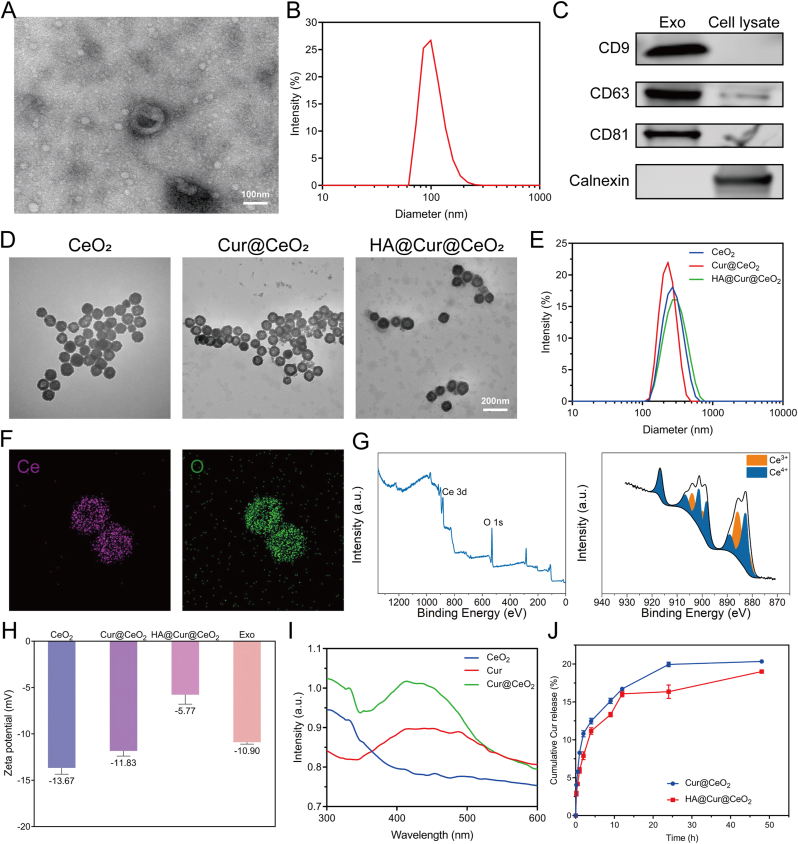
**Characterization of Exo and nanozymes.** (A) TEM image of Exo. (B) Particle size distributions of Exo. (C) Detection of exosomal markers (CD9, CD63 and CD81). (D) TEM images of prepared CeO_2_, Cur@CeO_2_ and HA@Cur@CeO_2_. (E) Particle size distributions of three nanozymes. (F) Elemental mapping images of HA@Cur@CeO_2_ nanozymes. (G) XPS spectra of HA@Cur@CeO_2_ nanozymes. (H) Zeta potential of Exo and nanozymes. (I) UV-vis spectra of Cur, CeO_2_, and Cur@CeO_2_. (J) The release curves of Cur from Cur@CeO_2_ and HA@Cur@CeO_2_.

Exo was isolated from the culture supernatant of DPSC and exhibited the characteristic “cup-shaped” morphology under TEM (Fig. 2A). Particle size analysis revealed that the mean diameter of Exo was 105 nm, with 99.27% of nanoparticles falling within the 30-200 nm range (Fig. 2B). Western blot results showed that Exo expressed specific surface markers CD9, CD63, and CD81, whereas not express Calnexin (Fig. 2C). These findings demonstrated that the isolated Exo conform to the latest MISEV2023 criteria established by the International Society for Extracellular Vesicles (ISEV) [27].

The synthesized nanozymes were then characterized. Representative TEM images illustrated the morphology of synthesized CeO_2_, Cur@CeO_2_ and HA@Cur@CeO_2_ nanoparticles (Fig. 2D). CeO_2_ nanoparticles displayed a uniform hollow spherical structure, and both Cur@CeO_2_ and HA@Cur@CeO_2_ maintained their regular spherical morphology and distinct hollow structure after the functionalization process. The presence of HA on the nanoparticle surface is anticipated to mitigate premature drug release and enhance nanoparticle biocompatibility [28]. The measured particle sizes were 265 nm for CeO_2_, 225 nm for Cur@CeO_2_, and 273 nm for HA@Cur@CeO_2_ (Fig. 2E). All three nanozymes exhibited excellent colloidal stability, as indicated by polydispersity index (PDI) < 0.3. Elemental mapping demonstrated the homogeneous distribution of Ce and O within the nanoparticles (Fig. 2F). Consistently, XPS analysis confirmed the presence of Ce and O elements, and the high-resolution Ce 3d spectrum revealed the coexistence of Ce^3+^ and Ce^4+^, which is essential for the ROS-scavenging activity of CeO_2_ nanozymes (Fig. 2G).

The surface properties and colloidal stability of the nanoparticles were further evaluated. Zeta potential measurements revealed that surface charges of −13.67 mV for CeO_2_, -11.83 mV for Cur@CeO_2_, -5.77 mV for HA@Cur@CeO_2_ and -10.90 mV for Exo (Fig. 2H). The surface potentials of both Exo and HA@Cur@CeO_2_ remained stable over a 6-day period (Fig. S1). The persistent negative surface charge contributed to the stability and dispersibility of Exo and HA@Cur@CeO_2_ in aqueous solutions, as evidenced by the narrow particle size distribution and consistently low PDI throughout the observation period (Figs. S2 and S3).

Successful Cur loading and HA modification were then verified. The UV-vis spectrum of Cur@CeO_2_ displayed the characteristic absorption peak of Cur, confirming successful drug loading (Fig. 2I). The encapsulation efficiency and drug loading capacity of Cur in CeO_2_ were 53.5 ± 2.8% and 33.3 ± 0.9%, respectively. The unique hollow structure of CeO_2_ nanoparticles facilitate superior drug loading, surpassing previously reported nanocarrier systems [29]. In addition, FTIR spectra further confirmed HA modification by the appearance of characteristic absorption bands of HA in HA@Cur@CeO_2_ (Fig. S4). XRD analysis verified the crystalline phase of CeO_2_ was preserved after Cur loading and HA modification (Fig. S5).

Finally, the release behavior of Cur was evaluated. As shown in Fig. 2J, both Cur@CeO_2_ and HA@Cur@CeO_2_ exhibited satisfactory stability with approximately 20% of Cur released within 48 h. Taken together, these results demonstrate the successful construction of HA@Cur@CeO_2_ nanozymes and support the potential of hollow mesoporous CeO_2_ nanozymes as a promising platform for drug delivery.

### 2.2 ROS scavenging ability of Exo and nanozymes in vitro

**Fig. 3:**
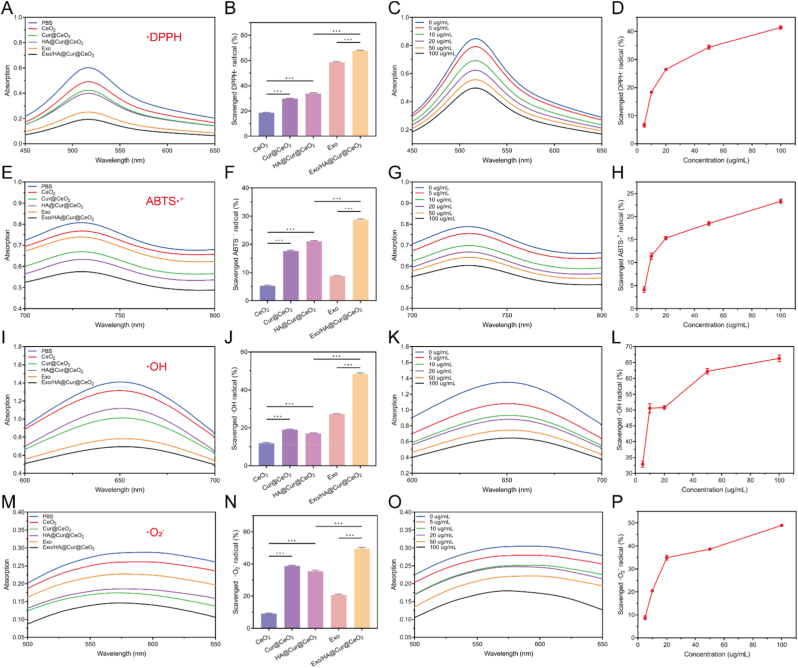
**ROS scavenging ability of Exo and nanozymes.** (A, B) DPPH• scavenging performance of Exo and nanozymes. (C, D) DPPH• scavenging performance of different concentrations of Exo/HA@Cur@CeO_2_. (E, F) ABTS•^+^ scavenging performance of Exo and nanozymes. (G, H) ABTS•^+^ scavenging performance of different concentrations of Exo/HA@Cur@CeO_2_. (I, J) •OH scavenging performance of Exo and nanozymes. (K, L) •OH scavenging performance of different concentrations of Exo/HA@Cur@CeO_2_. (M, N) •O_2_^−^ scavenging performance of Exo and nanozymes. (O, P) •O_2_^−^ -scavenging performance of different concentrations of Exo/HA@Cur@CeO_2_.

Eliminating excessive ROS and remodeling the inflammatory microenvironment are pivotal in OA treatment. The ROS scavenging capacities of Exo and nanozymes were assessed via their effects on DPPH• (Fig. 3AB), ABTS•^+^ (Fig. 3EF), •OH (Fig. 3IJ), and •O_2_^−^ (Fig. 3MN). As expected, the resulting HA@Cur@CeO_2_ demonstrated comparable ROS scavenging efficacy to Cur@CeO_2_, with both outperforming CeO_2_. This improvement is primarily ascribed to the incorporation of Cur, a natural antioxidant known to potentiate nanozyme-mediated free radical elimination [[29], [30], [31]].

Furthermore, the surface modification with HA as a “gatekeeper” facilitates Cur@CeO_2_'s selective binding to CD44 receptors on inflamed cells without affect its catalytic activity. This strategy leverages the upregulated CD44 expression in osteoarthritic chondrocytes and macrophages, enabling targeted delivery and more effective modulation of the inflammatory microenvironment [32,33]. Exo also exhibited evident antioxidant activity, which was consistent with previous studies [34,35]. Notably, Exo and HA@Cur@CeO_2_ nanozyme could function collaborate to clear the ROS. Subsequent analyses revealed that the scavenging of DPPH• (Fig. 3CD), ABTS•^+^ (Fig. 3GH), •OH (Fig. 3KL), •O_2_^−^ (Fig. 3OP), was concentration-dependent. Taken together, these findings provide foundational evidence for combined application of Exo and HA@Cur@CeO_2_ nanozyme in OA therapy to optimize therapeutic outcomes.

### 2.3 Characterization of the Exo/HA@Cur@CeO_2_/gel

**Fig. 4:**
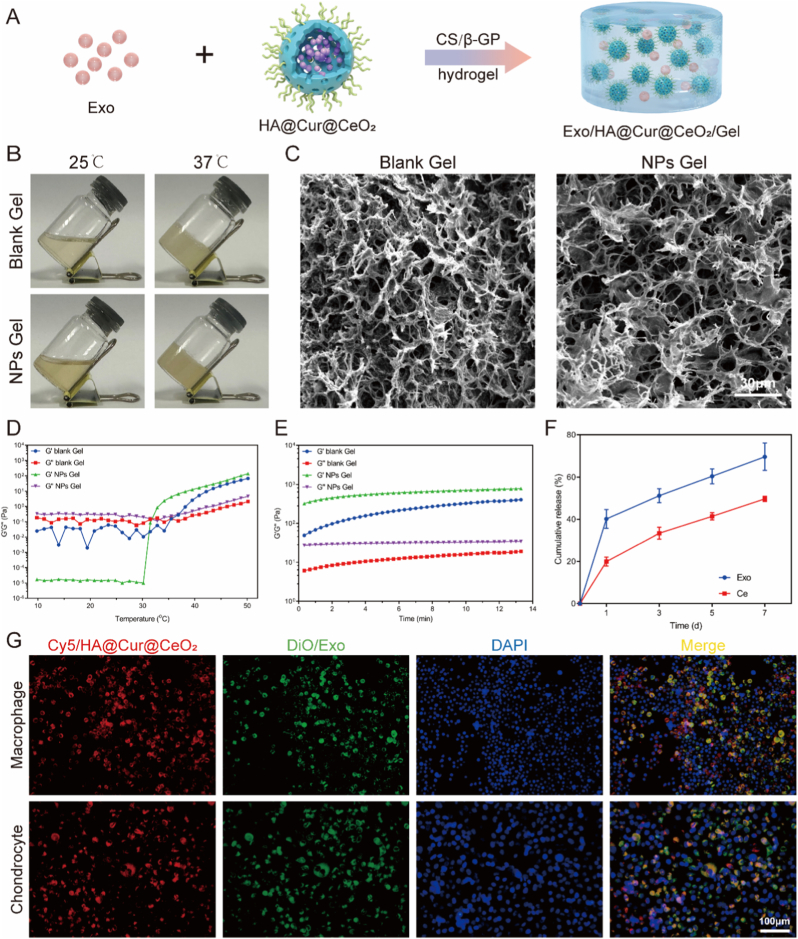
**Characterization of Exo/HA@Cur@CeO_2_/Gel and cellular uptake of nanoparticles.** (A) The protocol of Exo/HA@Cur@CeO_2_/Gel preparation. (B) Photographs of the hydrogels before (left) and after (right) gelation. (C) SEM images of hydrogels. Rheological properties of hydrogels with temperature (D) and time (E). (F) The release curves of Exo and Ce from the hydrogel under high-ROS conditions. (G) Cellular uptake of Exo and HA@Cur@CeO_2_.

Exo/HA@Cur@CeO_2_/Gel was prepared according to the procedures shown in Fig. 4A. At body temperature (37 °C), the mixed solution rapidly underwent a sol-gel transition, forming a stable hydrogel (Fig. 4B). SEM revealed a loose, porous microarchitecture characteristic of the hydrogels (Fig. 4C). Quantitative analysis showed a porosity of 23.9% for the blank gel and 17.3% for the Exo/HA@Cur@CeO_2_/Gel, indicating that Exo and HA@Cur@CeO_2_ were successfully incorporated into the porous matrix. Time- and temperature-dependent rheological analyses further demonstrated that incorporation of Exo and HA@Cur@CeO_2_ did not compromise the hydrogel's thermos-responsive gelation behavior or mechanical performance (Fig. 4DE). Notably, the storage modulus (G′) remained consistently higher than the loss modulus (G″), confirming the structural stability. These results suggested that the Exo/HA@Cur@CeO_2_/Gel can quickly switch from an injectable sol state to a stable non-flowing gel state in situ, which is expected to enhance local retention and reduce premature clearance driven by synovial fluid turnover and joint movement.

Given the elevated oxidative stress in OA joints, we further assessed hydrogel degradation under high-ROS conditions and compared it with that under physiological conditions. High ROS induced only a slight increase in the degradation rate, without significantly accelerating the overall degradation process (Fig. S6), indicating that the gel maintains sufficient structural integrity even in an oxidative microenvironment. The release kinetics of Exo and HA@Cur@CeO_2_ from the hydrogel under high-ROS conditions showed an initial burst release on day 1, followed by a sustained and gradual release over approximately one week (Fig. 4F). Moreover, compared with physiological conditions, OA-mimetic oxidative conditions moderately accelerated the release of both Exo and HA@Cur@CeO_2_ (Figs. S7 and S8). Collectively, these results confirm the successful integration of nanoparticles into the thermosensitive CS/β-GP hydrogel and support its capability for controlled, prolonged intra-articular delivery in vivo.

### 2.4 Cellular biocompatibility and uptake

The biocompatibility of biomaterials is crucial, as it directly interacts with several tissues within the joint, especially cartilage and synovium, following intra-articular injection [36]. Both Exo and CS/β-GP hydrogel have been extensively validated for their biocompatibility. Accordingly, we co-cultured varying concentrations of HA@Cur@CeO_2_ with chondrocytes and macrophages. The results demonstrated that HA@Cur@CeO_2_ exhibited negligible cytotoxicity across concentration range (0-100 μg/mL) after a 3-day incubation period (Fig. S9). A slight, statistically insignificant reduction in cell viability was observed at the concentration of 100 μg/mL. Besides, HA@Cur@CeO_2_ has already exhibited excellent ROS scavenging performance when the concentration reaches 50 μg/mL. Thus, to optimize therapeutic efficacy while minimizing potential cytotoxicity, 50 μg/mL was selected as the optimal concentration for subsequent experiments. Live/dead staining showed nearly all cells survived after 3 days of incubation (Figs. S10 and S11), further substantiating the excellent biocompatibility of HA@Cur@CeO_2_.

Efficient cellular internalization of therapeutic nanoparticles is critical for their intracellular delivery and subsequent pharmacological activity [37]. In this study, cellular uptake was assessed through incubating chondrocytes or macrophages with DiO-labeled Exo and Cy5-labeled HA@Cur@CeO_2_. After 24 h, pronounced green and red fluorescence signals were detected within the cytoplasm (Fig. 4G), confirming successful internalization of both nanoparticles by chondrocytes and macrophages, thereby facilitating their intended pharmacological actions. Flow cytometry further demonstrated a high degree of concurrent uptake of Exo and HA@Cur@CeO_2_ after 4 h of co-incubation, with 93.0% of macrophages and 98.9% of chondrocytes showing dual-positive signals (Fig. S12). To determine whether HA@Cur@CeO_2_ internalization is specifically mediated by CD44, HA competition assays were performed in both chondrocytes and macrophages. The presence of free HA markedly decreased intracellular Cy5 fluorescence (Figs. S13 and S14), suggesting that the cellular uptake of HA@Cur@CeO_2_ primarily depends on HA-CD44 interactions. Subsequent in vitro experiments were performed without the hydrogel to directly assess the intrinsic therapeutic functions of Exo and HA@Cur@CeO_2_.

### 2.5 Exo and HA@Cur@CeO_2_ inhibit macrophage inflammation

Macrophages play a pivotal role within the joint microenvironment by orchestrating both pro-inflammatory and anti-inflammatory signaling pathways [38]. In OA joints, elevated oxidative stress driven by ROS precipitates macrophage-mediated inflammation [22]. These activated macrophages, in turn, amplify ROS production and secrete pro-inflammatory mediators, forming a vicious circle [39]. Furthermore, the inflammatory milieu impairs the therapeutic efficacy of Exo [10]. Therefore, strategic modulation of the joint microenvironment is essential for effective OA intervention.

**Fig. 5:**
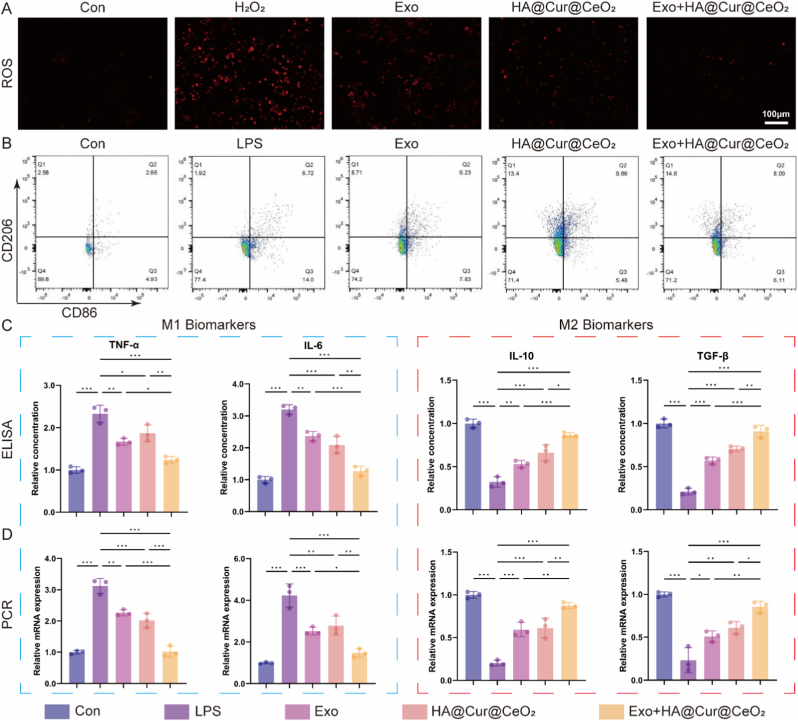
**Exo and HA@Cur@CeO_2_ inhibit macrophage inflammation.** (A) Representative fluorescent images of intracellular ROS levels. (B) Flow cytometry analysis of CD86^+^ and CD206^+^ cells. (C) ELISA analysis of concentrations of M1-associated cytokines (TNF-α and IL-6) and M2-associated cytokines (TGF-β and IL-10). (D) RT-qPCR analysis of the mRNA expression of M1-and M2-associated cytokines. All data are presented as means ± SD. n = 3 independent experiments. ∗P < 0.05, ∗∗P < 0.01, ∗∗∗P < 0.001.

The cooperative antioxidant and anti-inflammatory potential of Exo combined with HA@Cur@CeO_2_ nanozyme was systematically evaluated. Drawing upon the aforementioned results, it is reasonable to infer that both Exo and HA@Cur@CeO_2_ nanozyme confer substantial cytoprotective effects on RAW264.7 macrophages subjected to H_2_O_2_-induced oxidative stress. Consistent with expectations, treatment with either Exo or HA@Cur@CeO_2_ nanozyme led to a significant reduction in intracellular ROS levels, with their combined administration yielding an even more pronounced antioxidative effect (Fig. 5A, Fig. S15). Notably, the Exo + HA@Cur@CeO_2_ group exhibited ROS distributions closely resembling those of normal macrophages, underscoring the potent ROS scavenging capability achieved through their combined application.

A pivotal aspect of modulating the inflammatory microenvironment involves promoting macrophage polarization from the pro-inflammatory M1 phenotype to the anti-inflammatory M2 phenotype [40]. LPS was used to induce M1 polarization, and the effects of different treatments on polarization status were evaluated via flow cytometry. The results demonstrated a marked decrease in CD86 positive (M1) macrophages and a concomitant increase in CD206 positive (M2) macrophages following treatment with Exo, HA@Cur@CeO_2_, and their combination exhibiting the most pronounced effect (Fig. 5B, Fig. S16).

Furthermore, the secretion levels of proinflammatory cytokines predominantly synthesized by M1 macrophages, such as TNF-α and IL-6, along with anti-inflammatory mediators like IL-10 and TGF-β produced by M2 macrophages, were quantified via ELISA. After treatment, macrophages exhibited a marked reduction in M1-associated cytokine expression, whereas M2-associated cytokine expression was significantly elevated (Fig. 5C). Notably, the combined application of Exo and HA@Cur@CeO_2_ substantially augmented immunomodulatory efficacy. These pronounced immunomodulatory effects were further validated by RT-qPCR (Fig. 5D).

Collectively, these findings demonstrated that Exo and HA@Cur@CeO_2_ nanozyme cooperatively attenuate intracellular ROS and facilitate macrophage polarization towards the M2 phenotype, thereby remodeling the joint microenvironment and interrupting the inflammatory cascade. The resultant microenvironmental shift also mitigates chondrocyte inflammatory injury and paves the way for cartilage regeneration, underscoring the therapeutic promise of this combinatorial approach for OA management.

### 2.6 Exo and HA@Cur@CeO_2_ inhibit GPX4 and ALOX12-mediated chondrocyte ferroptosis

Chondrocytes, the only cells present in articular cartilage, are essential for preserving cartilage tissue integrity [41]. In OA, a pro-inflammatory milieu precipitates chondrocyte dysfunction and death, thereby disrupting the balance between extracellular matrix (ECM) synthesis and degradation, ultimately leading to cartilage degeneration [4,42]. Ferroptosis, an iron-dependent regulated cell death modality, has recently been identified as prevalent in osteoarthritic chondrocytes [43,44]. This process is typified by elevated ROS levels, iron overload, and increased lipid peroxidation [3,45]. Inhibiting chondrocyte ferroptosis could halt cartilage destruction and serve as another critical strategy for controlling OA progression.

**Fig. 6:**
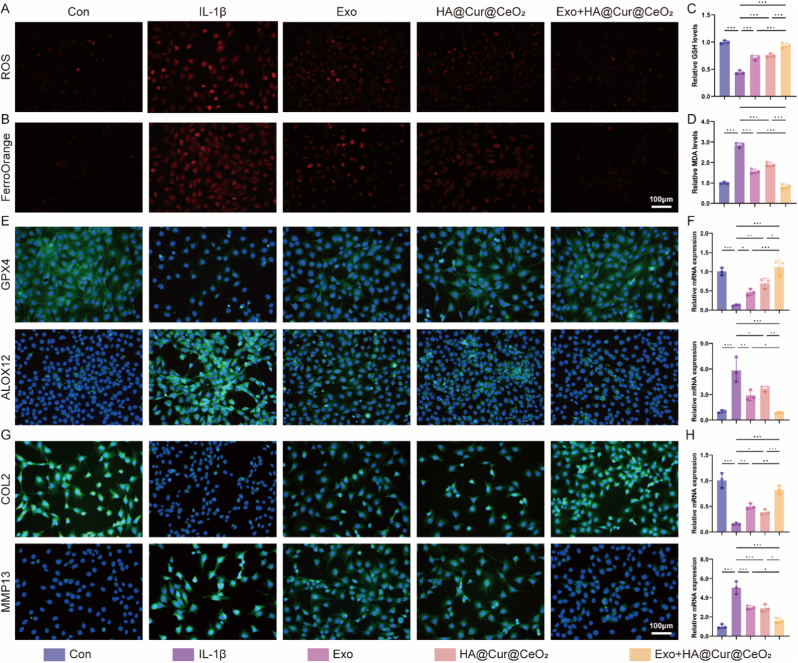
**Exo and HA@Cur@CeO_2_ inhibit GPX4 and ALOX12-mediated chondrocyte ferroptosis**. (A) Representative fluorescent images of intracellular ROS levels. (B) Representative fluorescent images of intracellular Fe^2+^ levels. (C) Detection of intracellular GSH levels. (D) Detection of intracellular MDA levels. (E) Representative immunofluorescence images of GPX4 and ALOX12. (F) RT-qPCR analysis of the mRNA expression of GPX4 and ALOX12. (G) Representative immunofluorescence images of COL2 and MMP13. (H) RT-qPCR analysis of the mRNA expression of COL2 and MMP13. All data are presented as means ± SD. n = 3 independent experiments. ∗P < 0.05, ∗∗P < 0.01, ∗∗∗P < 0.001.

To more accurately simulate the OA microenvironment in vitro, chondrocytes were treated with IL-1β to induce ferroptosis and ECM degradation. We evaluate the ROS scavenging capacity within IL-1β-induced chondrocytes following various treatments. Consistent with the above results, both Exo and HA@Cur@CeO_2_ nanozyme significantly attenuated ROS levels, with their combination exerting an improved effect (Fig. 6A, Fig. S17). IL-1β stimulation markedly elevated Fe^2+^ fluorescence intensity, which was substantially reduced following treatment with Exo, HA@Cur@CeO_2_, or their combination (Fig. 6B, Fig. S18). These treatments also mitigated the impact of IL-1β stimulation by restoring GSH levels (Fig. 6C) and reducing MDA levels (Fig. 6D). Notably, the Exo + HA@Cur@CeO_2_ group exhibited the most pronounced protective effects, underscoring its superior anti-ferroptotic activity. Overall, these results indicated that Exo + HA@Cur@CeO_2_ robustly inhibited chondrocyte ferroptosis by scavenging ROS, reducing Fe^2+^ accumulation and suppressing lipid peroxidation.

The underlying anti-ferroptotic mechanisms were further elucidated. GPX4 is a pivotal regulator of canonical ferroptosis inhibition, maintaining GSH redox homeostasis to prevent lipid peroxidation [46,47]. ALOX12, a recently characterized noncanonical ferroptosis mediator, catalyzes the peroxidation of polyunsaturated fatty acids in membrane phospholipids, promoting ferroptosis independently of GPX4 under ROS [48].

Immunofluorescence and semi-quantitative results revealed that Exo, HA@Cur@CeO_2_ and Exo + HA@Cur@CeO_2_ upregulated GPX4 expression while downregulating ALOX12 (Fig. 6E, Fig. S19). Specifically, Exo more effectively suppressed ALOX12, whereas HA@Cur@CeO_2_ more potently enhanced GPX4 expression. The combined treatment harnessed both mechanisms, conferring superior anti-ferroptotic efficacy. These results were validated through RT-qPCR quantification of GPX4 and ALOX12 mRNA expressions (Fig. 6F). To verify that the observed effects of HA@Cur@CeO_2_ were Cur-dependent, HA@CeO_2_ and HA@Cur@CeO_2_ were compared at identical concentrations. HA@CeO_2_ produced a modest increase in GPX4 and had little impact on ALOX12. In contrast, HA@Cur@CeO_2_ elicited markedly stronger GPX4 upregulation and robust ALOX12 downregulation (Fig. S20). This confirmed that GPX4 and ALOX12 modulation is primarily attributable to Cur release rather than the CeO_2_ scaffold alone.

Subsequently, the impact of Exo + HA@Cur@CeO_2_ on ECM homeostasis was evaluated via immunofluorescence and RT-qPCR. Col II, a principal ECM component, is predominantly degraded by MMP13 [49,50]. IL-1β exposure resulted in diminished Col II and elevated MMP13 expression, indicative of ECM degradation. Treatment with Exo, HA@Cur@CeO_2_ or their combination reversed these alterations, with the Exo + HA@Cur@CeO_2_ group exhibiting the most substantial restorative effect (Fig. 6G, Fig. S21). These results were further supported by RT-qPCR (Fig. 6H).

In summary, Exo and HA@Cur@CeO_2_ nanozyme complementarily inhibit chondrocyte ferroptosis and ECM degradation within the OA-associated pathological microenvironment. HA@Cur@CeO_2_, endowed intrinsic antioxidation ability properties, also exerts chondroprotective and anti-inflammatory effects via sustained Cur release. Cur has been demonstrated to counteract IL-1β-induced ECM breakdown and ferroptosis in chondrocytes [[51], [52], [53]]. Thus, the combined therapeutic strategy enhanced chondroprotection and impedes cartilage degeneration, offering significant potential for OA intervention.

### 2.7 Exploration of their potential therapeutic mechanisms in chondrocytes

**Fig. 7:**
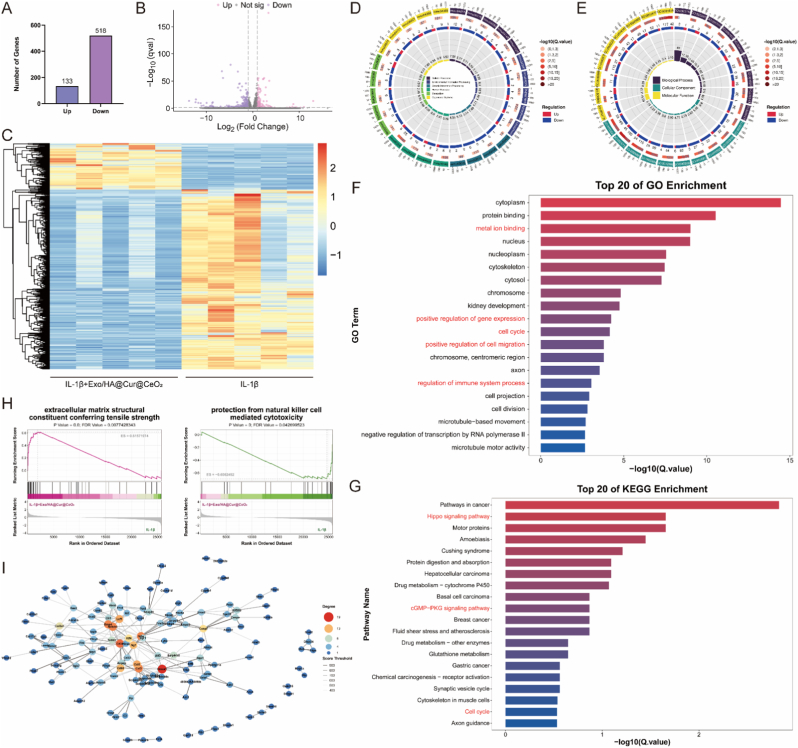
**Potential therapeutic mechanisms of Exo/HA@Cur@CeO_2_ in chondrocytes**. (A) Histogram showing the number of upregulated and downregulated genes (B) Volcano plot of DEGs. (C) Heatmap of DEGs. (D) Circos plot of enriched GO terms. (E) Circos plot of enriched KEGG pathways. (F) Enrichment results of top 20 GO terms. (G) Enrichment results of top 20 KEGG pathways. (H) GSEA enrichment results. (I) The PPI network of DEGs.

After confirming the protective effects of Exo/HA@Cur@CeO_2_ on OA in vitro, RNA sequencing was performed in chondrocytes treated with IL-1β or IL-1β + Exo/HA@Cur@CeO_2_ to explore the potential mechanisms of Exo/HA@Cur@CeO_2_ protection against OA. We identified 651 genes that were differentially expressed in chondrocytes after being treated with Exo/HA@Cur@CeO_2_. Of these, 133 genes were upregulated, and 518 genes were downregulated (Fig. 7A–C). GO analysis revealed that these DEGs were involved in metal ion binding, positive regulation of gene expression, cell cycle, positive regulation of cell migration and regulation of immune system process (Fig. 7D–F). KEGG pathway analysis indicated that Exo/HA@Cur@CeO_2_ regulated genes involved in Hippo signaling pathway, cGMP-PKG signaling pathway and cell cycle (Fig. 7E–G). GSEA results demonstrated that Exo/HA@Cur@CeO_2_ were enriched in ECM constituent conferring tensile strength and protection from natural killer cell mediated cytotoxicity (Fig. 7H). The PPI network was constructed to reveal the potential connection between DEGs (Fig. 7I). Finally, hub genes were identified (Fig. S22 and Table S1). Collectively, these findings indicated that Exo/HA@Cur@CeO_2_ markedly promoted cartilage ECM synthesis and efficiently regulated the immune microenvironment, thereby demonstrating substantial advantages in facilitating cartilage regeneration and delaying OA progression.

### 2.8 Exo/HA@Cur@CeO_2_/gel effectively delay OA progression in vivo

The encouraging in vitro efficacy of Exo + HA@Cur@CeO_2_ prompted us to further evaluate its therapeutic potential in vivo. Given the rapid clearance from the joint cavity, extending intra-articular retention time is critical for enhancing therapeutic outcome [54]. Therefore, we employed an injectable thermo-responsive CS/β-GP hydrogel, widely used as a delivery platform in OA, to encapsulate Exo and HA@Cur@CeO_2_ for sustained local release in the ACLT mice [23]. Mechanistically, the cationic nature of CS likely enables electrostatic interactions with the negatively charged Exo and HA@Cur@CeO_2_, which may reduce premature dispersion and help preserve their integrity [55]. In addition, the hydrogel's viscoelasticity and in situ gelation behavior promote localization at the injection site and resist rapid clearance driven by joint motion and synovial fluid turnover [56]. Collectively, these properties support prolonged joint retention and controlled release of both Exo and HA@Cur@CeO_2_, allowing long existence to exert their coordinated, multi-target effects on OA pathophysiology, as observed in our in vivo study.

**Fig. 8:**
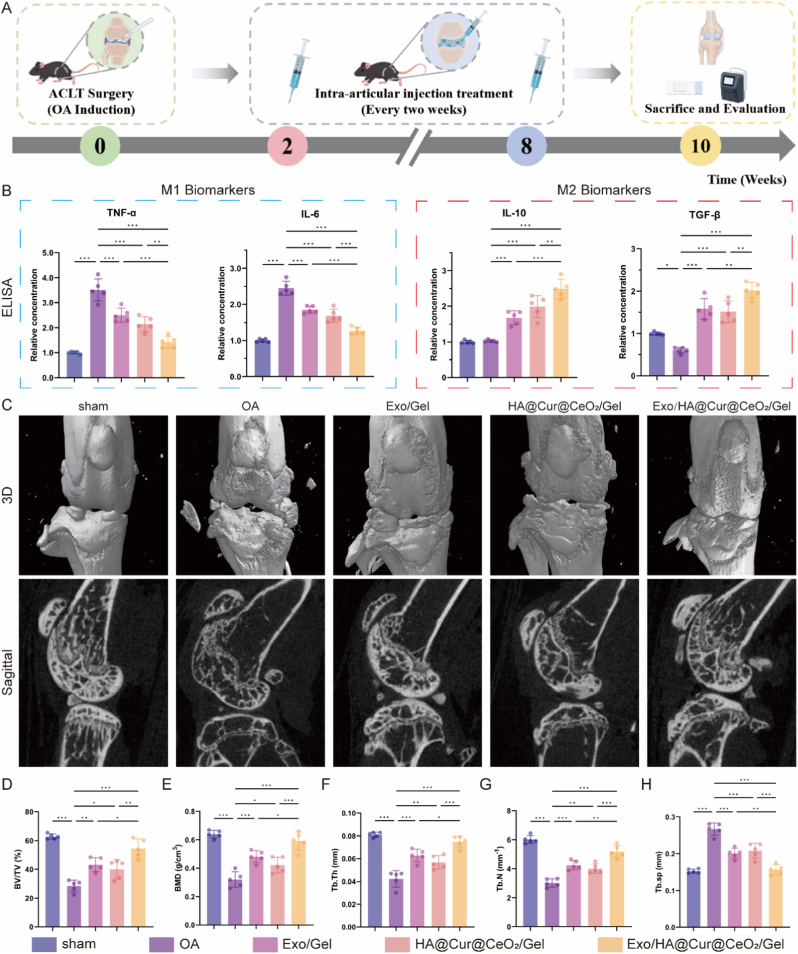
**Exo/HA@Cur@CeO_2_/Gel alleviates inflammation and subchondral bone remodeling in ACLT mice**. (A) The timeline of animal experiments. (B) ELISA analysis of concentrations of M1-associated proinflammatory cytokines (TNF-α and IL-6) and M2-associated anti-inflammatory cytokines (TGF-β and IL-10). (C) Representative micro-CT images for the 3D reconstruction of knee joints with sagittal views of the subchondral bone. Quantitative analysis of micro-CT parameters of tibial subchondral bone, including BV/TV (D), BMD (E), Tb.Th (F), Tb.N (G) and Tb.sp (H). All data are presented as means ± SD. n = 5 per group. ∗P < 0.05, ∗∗P < 0.01, ∗∗∗P < 0.001.

The animal experimental timeline is depicted in Fig. 8A. To assess microenvironmental remodeling performance, inflammatory cytokine levels in synovial fluid were quantified. Treatments with Exo/Gel, HA@Cur@CeO_2_/Gel and Exo/HA@Cur@CeO_2_/Gel significantly suppressed M1-associated proinflammatory cytokines (TNF-α and IL-6) and elevated the M2-associated anti-inflammatory cytokine (IL-10 and TGF-β) (Fig. 8B). These interventions promoted macrophage polarization towards the M2 phenotype, conferring excellent anti-inflammatory effects in vivo.

Micro-CT analysis was performed to evaluate subchondral bone preservation. The 3D reconstructed images and sagittal images showed that all treatment groups attenuated osteophyte formation and maintained subchondral bone microarchitecture (Fig. 8C). Quantitative parameters, including increased BV/TV, BMD, Tb.Th, Tb.N (Fig. 8D–G), and decreased Tb.Sp (Fig. 8H), further substantiated these protective effects. The Exo/HA@Cur@CeO_2_/Gel group exhibited bone parameters closely mirroring those of the sham group, highlighting its efficacy in mitigating aberrant bone remodeling.

**Fig. 9:**
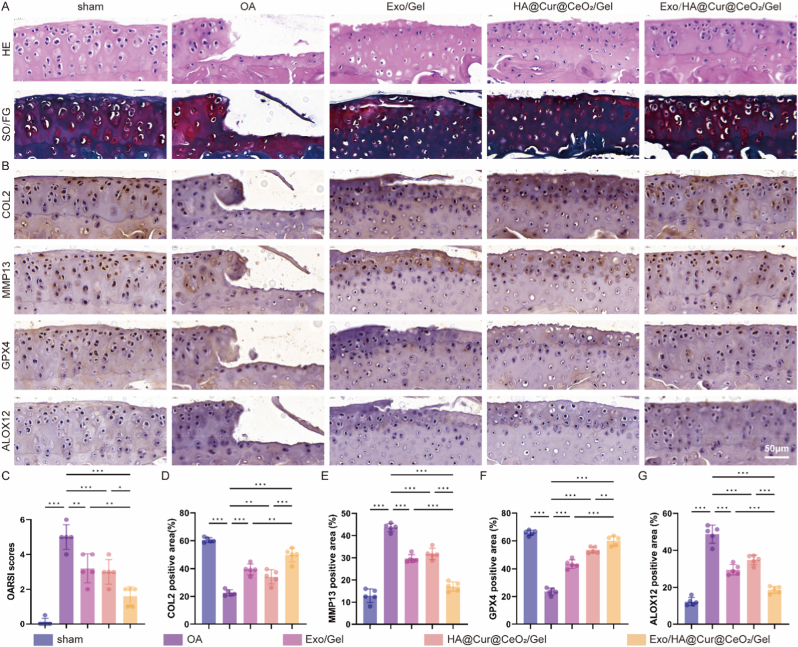
**Exo/HA@Cur@CeO_2_/Gel inhibits chondrocyte ferroptosis and cartilage degeneration in ACLT mice**. (A) Representative images of HE and SO/FG staining. (B) Representative IHC images of COL2, MMP13, GPX4 and ALOX12. (C) OARSI scores of SO/FG staining results. Semi-quantitative analysis of IHC staining results for COL2 (D), MMP13 (E), GPX4 (F) and ALOX12 (G). All data are presented as means ± SD. n = 5 per group. ∗P < 0.05, ∗∗P < 0.01, ∗∗∗P < 0.001.

HE and safranin O-fast green staining were conducted to evaluate the chondroprotective of Exo/HA@Cur@CeO_2_/Gel on articular cartilage. As illustrated in Fig. 9A, the OA group exhibited pronounced cartilage surface irregularities, extensive matrix erosion, chondrocyte depletion, and severe structural defects. Administration of Exo/Gel, HA@Cur@CeO_2_/Gel and Exo/HA@Cur@CeO_2_/Gel markedly mitigated cartilage degeneration, as evidenced by reduced OARSI scores (Fig. 9C). Fortunately, Exo/HA@Cur@CeO_2_/Gel-treated mice displayed cartilage morphology most comparable to the sham group, underscoring its superior chondroprotective efficacy. Immunohistochemical staining results are presented in Fig. 9B. The OA group exhibited a marked reduction in Col II expression within the ECM; however, this expression was significantly restored following treatment with Exo/Gel, HA@Cur@CeO_2_/Gel and Exo/HA@Cur@CeO_2_/Gel (Fig. 9D). These findings were consistent with the results observed in the HE and safranin O-fast green staining. Furthermore, MMP13 expression was substantially downregulated in the treated groups relative to OA controls (Fig. 9E). Regarding ferroptosis markers, ALOX12 was upregulated and GPX4 downregulated in OA joints, alterations that were reversed following treatment (Fig. 9FG). Exo/Gel and HA@Cur@CeO_2_/Gel demonstrated specificity in modulating ALOX12 and GPX4, respectively, while Exo/HA@Cur@CeO_2_/Gel exerted the most pronounced regulatory effects, achieving expression profiles akin to the sham group. These results indicated that Exo/HA@Cur@CeO_2_/Gel effectively prevented ECM degradation and inhibited chondrocyte ferroptosis, thereby preserving cartilage structural integrity. Importantly, while ferroptosis inhibition likely contributes to these benefits, the observed joint-level protection is most likely mediated by coordinated multi-target effects, including redox rebalancing, inflammatory microenvironment remodeling, and ECM homeostasis preservation.

A critical prerequisite for effective OA therapy is the ability of therapeutic components to penetrate the cartilage ECM and reach resident chondrocytes [57]. In OA, pathological disruption of the collagen-proteoglycan network increases matrix porosity, potentially permitting the selective entry of nanoparticles ranging from 100 to 300 nm [58]. This size compatibility may enable both Exo and HA@Cur@CeO_2_ to permeate the compromised ECM with reduced entrapment, thereby facilitating their enrichment within the lesion area. Furthermore, the CS/β-GP hydrogel primarily serves as an intra-articular depot that prolongs joint residence and provides sustained release of Exo and HA@Cur@CeO_2_, helping maintain a local concentration gradient that can support continuous diffusion toward deeper cartilage zones over time [59,60]. Moreover, the therapeutic effect of HA@Cur@CeO_2_ is partially mediated by the sustained release of Cur. As a small polyphenolic molecule, Cur is expected to diffuse more readily through the ECM to reach deep-zone chondrocytes [61]. Future studies will further quantify and optimize the cartilage penetration ability of these released components.

At present, clinical OA treatments such as corticosteroids and HA injections mainly provide temporary symptomatic relief but lack disease-modifying capabilities. Their efficacy is further limited by rapid clearance from the synovial cavity, necessitating repeated injections. In contrast, our Exo/HA@Cur@CeO_2_/Gel platform offers several distinct advantages. Rather than targeting a single pathway, our platform employs a multi-targeted approach that simultaneously mitigates oxidative stress, synovial inflammation, and chondrocyte ferroptosis through the complementary action of Exo and HA@Cur@CeO_2_. Moreover, the CS/β-GP hydrogel provides enables prolonged intra-articular retention and controlled release over weeks, potentially reducing the frequency of injections. By shifting the therapeutic focus from symptom control to modulation of key OA pathophysiological processes, this platform may offer a potential disease-modifying strategy with significant translational potential.

In vivo biocompatibility was evaluated. HA@Cur@CeO_2_ exhibited low hemolytic rate (<5%) across a range of concentrations (Fig. S23), suggesting a minimal potential risk of hemolysis. Serum biochemical analyses indicated that treatment with Exo/HA@Cur@CeO_2_/Gel did not alter levels of alanine aminotransferase (ALT), aspartate aminotransferase (AST), blood urea nitrogen (BUN), or creatinine (CREA) (Fig. S24). Additionally, HE staining of major organs revealed preserved normal histological architecture with no evidence of pathological changes (Fig. S25). Collectively, no significant hemolysis, major organ toxicity, or histopathological abnormalities were observed (Figs. S23–S25), confirming the favorable safety profile of the platform.

Although our study demonstrated encouraging therapeutic outcomes and excellent short-term biocompatibility, certain limitations persist. First, while each component of Exo/HA@Cur@CeO_2_/Gel has been widely reported to exhibit favorable biocompatibility, the potential cumulative effects associated with repeated administration over extended periods have not yet been fully elucidated. Second, therapeutic efficacy may attenuate over time, and the durability of the sustained benefit provided by our platform remains to be determined. Accordingly, future studies will include longer follow-up periods, comprehensive and systematic safety and efficacy evaluations, detailed biodistribution and clearance assessments, and validation in large-animal OA models. Third, further steps toward clinical translation will require scalable and standardized manufacturing of both Exo and nanozymes, together with robust quality control to ensure batch-to-batch consistency. Fourth, although our results demonstrated complementary modulation of GPX4-and ALOX12-related ferroptotic pathways by Exo and HA@Cur@CeO_2_, we did not perform rescue experiments to determine whether inhibiting GPX4 or activating ALOX12 reverses the protective effects of HA@Cur@CeO_2_ or Exo, respectively. Future studies will incorporate such rescue approaches (e.g., RSL3 for GPX4 inhibition and genetic activation of ALOX12) to confirm pathway dependence.

Overall, these findings indicate that Exo/HA@Cur@CeO_2_/Gel robustly suppresses local inflammation, chondrocyte ferroptosis, cartilage degeneration and subchondral bone remodeling, collectively contribute to retarding OA progression. Notably, the Exo/HA@Cur@CeO_2_/Gel conferred the most pronounced therapeutic benefit among all treatments. This superior outcome is likely attributable to the HA@Cur@CeO_2_ nanozyme, which may help maintain a more favorable microenvironment for Exo function. Specifically, HA@Cur@CeO_2_ nanozyme facilitates ROS scavenging and promotes macrophage polarization from the pro-inflammatory M1 phenotype to the anti-inflammatory M2 phenotype, thereby reprogramming the joint inflammatory milieu in OA. Within this modulated environment, Exo may encounter a reduced immunomodulatory burden, potentially enabling a greater proportion of their bioactivity to be directed toward direct chondroprotection and ECM homeostasis. Furthermore, the sustained release of Cur from HA@Cur@CeO_2_ may further enhance therapeutic efficacy. Previous studies have shown that Cur markedly attenuated cartilage destruction and synovitis [62,63]. However, its limited bioavailability has hindered intra-articular application, a challenge that our nanozyme-based delivery system is designed to address [64]. Additionally, Cur can serve as an adjuvant to MSC-derived exosomes, augmenting their bioactivity, particularly under inflammatory conditions, and enhancing overall therapeutic outcomes [65,66]. Regarding ferroptosis, the pronounced protective effects likely arise from the complementary modulation of two key regulatory nodes: Exo primarily suppresses ALOX12, whereas HA@Cur@CeO_2_ effectively upregulates GPX4, thereby more comprehensively interrupting the ferroptotic cascade and contributing to chondroprotection. By leveraging the complementary advantages of Exo and HA@Cur@CeO_2_, Exo/HA@Cur@CeO_2_/Gel therefore possesses the ability to simultaneously address multiple pathological mechanisms of OA, potentially attenuating disease progression. Coupled with its excellent biocompatibility, our study presents a novel and promising combinatorial strategy for OA treatment, laying the foundation for future clinical translation.

## 3 Conclusions

In summary, we have developed a novel combinatorial therapeutic platform that addresses multiple pathophysiological processes of OA. This approach integrates DPSC-derived exosomes with Cur-loaded hollow mesoporous CeO_2_ to construct HA@Cur@CeO_2_ nanozymes, encapsulated within a thermosensitive CS/β-GP hydrogel, to provide sustained therapeutic effects. The resulting nanozymes exhibited potent ROS scavenging activity, enabled controlled Cur release, and demonstrated targeted delivery to inflamed cells under OA conditions, significantly improving therapeutic outcomes. In vitro studies demonstrated that the combination of Exo and HA@Cur@CeO_2_ nanozymes cooperatively reduced intracellular ROS, promoted macrophage polarization toward the anti-inflammatory M2 phenotype, and remodeled the OA microenvironment by disrupting the inflammatory cascade. In chondrocytes, Exo and HA@Cur@CeO_2_ specifically modulated ALOX12-and GPX4-mediated ferroptosis, respectively, with their combination yielding superior anti-ferroptotic effects. For in vivo assessment, Exo and HA@Cur@CeO_2_ were encapsulated within CS/β-GP hydrogel for sustained release. The Exo/HA@Cur@CeO_2_/Gel formulation significantly suppressed local inflammation, chondrocyte ferroptosis, cartilage degeneration and subchondral bone remodeling, thereby decelerating OA progression. Collectively, our combinatorial therapeutic approach leverages the advantages of Exo and HA@Cur@CeO_2_ to concurrently address multiple OA pathophysiological processes, offering a highly effective and comprehensive strategy. With excellent biocompatibility, this innovative combinatorial therapeutic strategy represents a comprehensive approach for enhancing Exo efficacy in OA treatment with promising translational potential.

## 4 Experimental section

### 4.1 Isolation of Exo

The DPSCs were procured from the Hebei Taihe Yunshan Biotechnology Co., Ltd. (China). Exo were isolated from the culture supernatants via differential centrifugation according to established protocols [25,67]. The harvested Exo were resuspended in PBS and stored at −80 °C for further experiments.

### 4.2 Synthesis of hollow mesoporous CeO_2_ nanoparticles

Hollow mesoporous CeO_2_ nanoparticles were synthesized using a wet chemical method. In brief, cerium nitrate hexahydrate (0.5 g) and PVP K30 (0.2 g) were dissolved in 15 mL of ethylene glycol. Subsequently, 1 mL of 1 M hydrochloric acid aqueous solution was added under vigorous agitation. After stirring for 30 min, the clear solution was transferred into a Teflon-lined autoclave and heated for 3 h at 160 °C. After cooling to room temperature, the gray nanoparticles were collected and washed three times with deionized water at 10000 rpm for 20 min.

### 4.3 Synthesis of HA@Cur@CeO_2_

A total of 100 mg of CeO_2_ was dispersed in 10 mL of deionized water. Cur was dissolved in DMSO to achieve a final concentration of 25 mM. The Cur solution was then dropwise added to the CeO_2_ suspension. After stirring for 12 h, HA was introduced at a mass ratio of 1:4, and then stirred for an additional 12 h. The resulting product was washed three times alternately with water and ethanol at 10000 rpm for 20 min. The final HA@Cur@CeO_2_ nanoparticles were dispersed in deionized water.

### 4.4 Preparation of Exo/HA@Cur@CeO_2_/gel

Chitosan (CS, 90% deacetylation degree) was dissolved in a 1.2 wt% lactic acid solution to obtain a 1.5 wt% CS solution. β-Glycerophosphate (β-GP) was dissolved in 0.1 M NaHCO_3_ to prepare a 6 wt% β-GP solution. CS solution and β-GP solution were mixed thoroughly at a 1:1 vol ratio, followed by the rapid addition of 100 μL of 1 mg/mL HA@Cur@CeO_2_ solution and 10^10^ Exo. The above mixture solution was incubated at 37 °C for 10 min to yield a thermosensitive hydrogel loaded with HA@Cur@CeO_2_ and Exo.

### 4.5 Characterization

Transmission electron microscopy (TEM, Hitachi HT7800) was employed to characterize the morphology of nanoparticles, whereas dynamic light scattering (DLS, Malvern Zetasizer Nano ZS) was utilized to determine particle size, size distribution, and zeta potential. Elemental mapping (JEOL JEM-F200) was performed to analyze the elemental distribution within the nanoparticles. X-ray photoelectron spectroscopy (XPS, Thermo Scientific K-Alpha) was used to determine the surface elemental composition and valence states. X-ray diffraction (XRD, Rigaku SmartLab SE) was performed to examine the crystalline phase of the nanoparticles. Fourier-transform infrared spectroscopy (FTIR, Thermo Scientific Nicolet iS20) was used to verify HA modification by identifying characteristic surface functional groups, and UV-vis spectroscopy was employed to confirm Cur loading. Western blotting was performed to detect the exosomal markers CD9, CD63, and CD81, and Calnexin in Exo and cell lysate.

Scanning electron microscopy (SEM, Hitachi Regulus 8100) facilitated the observation of hydrogel surface morphology, and ImageJ software was utilized to perform quantitative porosity analysis on the SEM images. A rheometer (TA Discovery HR-2) was used to evaluate the rheological properties as a function of time and temperature. The in vitro degradation profile was determined by the mass loss method, where hydrogel samples were incubated in either PBS (pH 7.4) or PBS supplemented with 100 μM H_2_O_2_ at 37 °C to simulate physiological and high-ROS microenvironments, respectively.

### 4.6 Drug loading and release

To assess drug loading, Cur@CeO_2_ was dispersed in ethanol and then centrifuged at 12000 rpm for 15 min. The content of Cur in the supernatant was analyzed by UV-vis spectrophotometer at 430 nm. Encapsulation efficiency and loading capacity of Cur were calculated by the following formula:

$$E\text{ncapsulation}\;\text{efficiency}\;\left(\%\right)=\frac{Total\;Cur-unencapsulated\;Cur\;}{Total\;Cur}\times 100$$

$$\text{Loading}\;\text{capacity}\;\left(\%\right)=\frac{Weight\;of\;loaded\;Cur\;in\;nanoparticles}{Weight\;of\;nanoparticles\;}\times 100$$

To assess drug release, Cur-loaded nanoparticle suspensions were sealed into a dialysis bag (M_W_: 10 kDa), and immersed in pH 7.4 PBS at 37 °C for 48 h. At predetermined time points, 1 mL of release medium was collected, and replaced with equivalent volumes of fresh PBS. The released content of Cur was measured by UV-vis spectrophotometer.

### 4.7 DPPH• elimination

2,2-diphenyl-1-picrylhydrazyl (DPPH) was dissolved in ethanol to prepare the working solution. Nanoparticle samples were added into DPPH solution and incubated in the dark for 30 min. The absorbance was assessed by UV-vis spectrophotometer at 517 nm.

### 4.8 ABTS•^+^ elimination

2,2′-azino-bis(3-ethylbenzothiazoline-6-sulfonic acid) (ABTS) was incubated with potassium persulfate solution for 16 h to obtain the working solution. Nanoparticle samples were added into ABTS solution and incubated in the dark for 10 min. The absorbance was detected at 734 nm.

### 4.9 •OH elimination

FeSO_4_ was incubated with H_2_O_2_ for 10 min to generate hydroxyl radical (•OH) solution by Fenton reaction. Nanoparticle samples were added into •OH solution for 30 min, followed by the addition of salicylic acid for a further 10 min. The absorbance was recorded at 510 nm.

### 4.10 •O_2_^−^ elimination

NADH, PMS, and NBT were mixed to prepare superoxide anion radical (•O_2_^−^) solution. Then, nanoparticle samples were added into •O_2_^−^ solution and illuminated under ultraviolet radiation for 10 min. The absorbance was measured at 560 nm.

### 4.11 Cell culture

The murine chondrocytes ATDC5 and macrophages RAW264.7 cell line were purchased from Zhong Qiao Xin Zhou Biotechnology Co., Ltd. (Shanghai, China). The cells were cultured in different basic media (Chondrocytes: DMEM/F12; RAW264.7: DMEM) (Gibco, USA) supplemented with 10% fetal bovine serum (FBS) (Gibco, USA) and 1% penicillin/streptomycin (Solarbio, China) at 37 °C with 5% CO_2_. Unless otherwise specified, all cellular functional assays employed Exo at a concentration of 1 × 10^9^ particles/mL and HA@Cur@CeO_2_ at 50 μg/mL.

### 4.12 In vitro biocompatibility

Cells were co-cultured with 5, 10, 20, 50 or 100 μg/mL HA@Cur@CeO_2_ for 72 h. Cell viability was evaluated using the CCK-8 assay (Zeta Life, USA) by adding the reagent and incubating for 2 h, followed by measurement of absorbance at 450 nm. For the Live/Dead staining assay, cells were incubated with Calcein-AM/PI (Beyotime, China) for 30 min and then observed under a fluorescence microscope (Olympus, Japan).

### 4.13 Reactive oxygen species (ROS) assay

Intracellular ROS content was measured by ROS Fluorometric Assay Kit (Elabscience, China). The cells to be tested were incubated with 5 μM/L DCFH-DA for 30 min at 37 °C, and then observed under a fluorescence microscopy.

### 4.14 Intracellular iron assay

Intracellular Fe^2+^ was assessed by FerroOrange (Dojindo, Japan). The cells to be tested were incubated with 1 μM/L FerroOrange in serum-free medium for 30 min at 37 °C, and then observed under a fluorescence microscopy.

### 4.15 Malonaldehyde (MDA) assay

Intracellular MDA content was measured by MDA Fluorometric Assay Kit (Elabscience, China). The cells to be tested were lysed to prepare supernatants. The supernatants were reacted with thiobarbituric acid (TBA) for 40 min at 100 °C, and then examined by a fluorescence microplate reader at Ex/Em = 520/550 nm.

### 4.16 Glutathione (GSH) assay

Intracellular GSH content was measured by GSH Assay Kit (Beyotime, China). The cells to be tested were lysed to prepare supernatants. The supernatants were reacted with GSH assay mix for 25 min at 25 °C, and then examined by a microplate reader at 412 nm.

### 4.17 Immunofluorescence

The cells were fixed in 4% paraformaldehyde and permeabilized with 0.1% Triton X-100. After blocking with 5% goat serum for 1 h, the chondrocytes were incubated with the corresponding primary antibodies: anti-Col II (ab188570, Abcam), anti-MMP13 (ab39012, Abcam), anti-GPX4 (67763-1-Ig, Proteintech) and anti-ALOX12 (A14703, ABclonal) overnight at 4 °C, followed by incubation with Alexa Fluor 488-conjugated anti-rabbit or anti-mouse secondary antibodies (Abbkine, China). Nuclei were stained with DAPI for 15 min and then observed under a fluorescence microscopy.

### 4.18 Macrophages polarization

RAW264.7 cells were stimulated with 100 ng/mL LPS (Sigma, USA) for 24 h to induce M1 macrophage polarization, followed by incubation with various nanoparticle formulations for 24 h. After washing with PBS for 3 times, the cells were stained with Alexa Fluor 647-CD206 (568808, BD Pharmingen) and PE-CD86 (A27137, Abclonal) in the dark for 30 min. Subsequently, the cells were washed, resuspended, and immediately analyzed by flow cytometry (Beckman, CytoFlex, USA). Additionally, expression levels of M1-associated cytokines (TNF-α and IL-6) and M2-associated cytokines (TGF-β and IL-10) were quantified by ELISA and RT-qPCR.

### 4.19 RNA sequencing analysis

ATDC5 cells were incubated with IL-1β or IL-1β + Exo/HA@Cur@CeO_2_ for 24 h. Total RNA was isolated using Trizol reagent (Thermo, USA) and then subjected to high-throughput RNA sequencing by LC Bio (Hangzhou, China) utilizing the Illumina platform. Differentially expressed genes (DEGs) were defined by log_2_|(fold change)| >1 and q value < 0.05. Functional annotation of DEGs was performed through Gene Ontology (GO), Kyoto Encyclopedia of Genes and Genomes (KEGG) pathway analysis, and Gene Set Enrichment Analysis (GSEA). Additionally, protein-protein interaction (PPI) networks were constructed, and hub genes were identified.

### 4.20 Animal studies

The animal study protocol received approval from the institutional animal ethics committee. Twelve-week-old male C57BL/6 mice were obtained from Vitalriver (Beijing, China) and randomly assigned to the following groups (n = 5 per group): sham, OA, Exo/Gel, HA@Cur@CeO_2_/Gel, and Exo/HA@Cur@CeO_2_/Gel. The sample size was determined based on previous studies [67]. The sham group underwent only a skin and subcutaneous tissue incision, whereas OA was induced in the other groups via anterior cruciate ligament transection (ACLT) [68]. Two weeks after ACLT, mice received unilateral intra-articular injections of 10 μL of the respective formulations every two weeks for a total duration of 8 weeks. Mice were sacrificed at 2 weeks after the final injection, and the knee joints samples were harvested.

### 4.21 Micro-CT analysis

Knee joints samples were scanned using a high-resolution micro-CT scanner (Bruker, Belgium) at a voxel size of 9 μm. The acquired images were used for three-dimensional reconstruction, and then subchondral bone microarchitecture parameters of tibial plateau were assessed. Parameters evaluated included bone volume/tissue volume (BV/TV), trabecular thickness (Tb.Th), trabecular number (Tb.N), trabecular separation (Tb.Sp) and bone mineral density (BMD).

### 4.22 Histological and immunohistochemistry (IHC) analysis

Following micro-CT scanning, the samples were fixed in 4% paraformaldehyde for 24 h and decalcified in 10% EDTA for 4 weeks. The samples were then paraffin-embedded and sectioned at a thickness of 4 μm. For histological analysis, sections were stained with hematoxylin-eosin (HE), and safranin O-fast green (SO/FG). The Osteoarthritis Research Society International (OARSI) scores were calculated according to previous study [69]. IHC stanning of Col II (GTX100829, GeneTex), MMP13 (GTX100665, GeneTex), GPX4 (30388-1-AP, Proteintech), and ALOX12 (A14703, ABclonal) were also conducted.

### 4.23 ELISA

Synovial tissues and synovial fluid were collected and then homogenized in RIPA buffer. The concentrations of TNF-α, IL-6, IL-10 and TGF-β in the supernatants were measured using commercial ELISA kits and normalized to total protein content determined by a BCA assay.

### 4.24 In vivo biocompatibility

At the end of animal experiments, when euthanizing the mice, major organs including hearts, livers, spleens, lungs, and renal tissues were also harvested and subjected to HE staining for histopathological evaluation. Additionally, blood samples were obtained for biochemical tests, including alanine aminotransferase (ALT), aspartate aminotransferase (AST), blood urea nitrogen (BUN), and creatinine (CREA).

### 4.25 Hemolysis test

Mouse red blood cell suspensions were prepared and incubated with water (positive control), normal saline (negative control), and different concentrations of HA@Cur@CeO_2_ (5, 10, 20, 50 or 100 μg/mL) at 37 °C for 3 h. Following incubation, the mixtures were centrifuged at 2500 rpm for 10 min, and the absorbance of the supernatants was measured to calculate hemolysis rates.

### 4.26 Statistical analysis

The data were presented as mean ± standard deviations (SD). Normality was assessed using the Shapiro-Wilk test. Student's t-test was used for comparisons between two groups. One-way analysis of variance (ANOVA) and Tukey post hoc test were for comparisons between multiple groups. Statistical analysis was performed using R software (version 4.4), with a P-value of less than <0.05 considered statistically significant.

## Institutional review board statement

Our study was approved by the local ethical committee (Z2024-007-1).

## Declaration of generative AI and AI-assisted technologies in the writing process

During the preparation of this work the authors used Dochero (www.dochero.ai) in order to polish the language. After using this tool, the authors have reviewed and edited the content as needed and take full responsibility for the content of the publication.

## Funding

Our work was supported by the Postdoctoral Fund of 10.13039/501100012505Hebei Medical University, 10.13039/501100001809National Natural Science Foundation of China (81873983), 10.13039/501100003787Natural Science Foundation of Hebei Province (H2022206534, H2021206162), Hebei Province Science and Technology Support Program (252W7710D), Graduate Innovation Project of Hebei Province (CXZZBS2024128) and Natural Science Foundation of 10.13039/501100012505Hebei Medical University for Outstanding Doctoral Student (YBKJ202503).

## CRediT authorship contribution statement

**Chenyue Xu:** Conceptualization, Funding acquisition, Investigation, Methodology, Project administration, Visualization, Writing – original draft. **Zhengyi Ni:** Conceptualization, Investigation, Methodology, Project administration, Writing – original draft. **Qi Wang:** Investigation, Methodology, Project administration, Writing – original draft. **Handi Li:** Investigation, Methodology, Project administration, Visualization. **Lie Wu:** Investigation, Methodology, Project administration, Visualization. **Yuhang Shi:** Investigation, Methodology, Project administration, Visualization. **Xiaobo Chen:** Methodology, Project administration, Visualization. **Ziyi Li:** Methodology, Project administration. **Huijun Kang:** Conceptualization, Methodology, Project administration, Supervision, Validation, Writing – review & editing. **Yanxin Liu:** Conceptualization, Investigation, Methodology, Project administration, Writing – review & editing. **Zeyu Liu:** Conceptualization, Investigation, Methodology, Project administration, Supervision, Writing – review & editing. **Fei Wang:** Conceptualization, Funding acquisition, Investigation, Project administration, Supervision, Validation, Writing – review & editing.

## Declaration of competing interest

The authors declare the following financial interests/personal relationships which may be considered as potential competing interests:Fei Wang reports financial support was provided by National Natural Science Foundation of China. Fei Wang reports financial support was provided by Natural Science Foundation of Hebei Province. Fei Wang reports financial support was provided by Hebei Province Science and Technology Support Program. Chenyue Xu reports financial support was provided by Postdoctoral Fund of Hebei Medical University. Chenyue Xu reports was provided by Graduate Innovation Project of Hebei Province. Chenyue Xu reports was provided by Natural Science Foundation of Hebei Medical University for Outstanding Doctoral Student. If there are other authors, they declare that they have no known competing financial interests or personal relationships that could have appeared to influence the work reported in this paper.

## Data Availability

Data will be made available on request.
