# Supplementary material for: Enhancing exosomes efficacy with engineered nanozymes for a multi-targeted combination strategy in osteoarthritis treatment
Source: Mater Today Bio. 2026 Apr 12;38:103118. doi: 10.1016/j.mtbio.2026.103118 (PMC13101615; doi:10.1016/j.mtbio.2026.103118)
Supplement: Multimedia component 1 [file mmc1.docx]

**Table S1.** Degree of top 10 hub genes.

| **Rank** | **Gene name** | **Biological functions** | **Score** |
| --- | --- | --- | --- |
| 1 | Bmp4 | TGF-β/BMP family morphogen; regulates osteogenesis, chondrogenesis, and ECM synthesis | 146 |
| 2 | Cd34 | Endothelial and progenitor cell marker; mediates cell migration, angiogenesis, and tissue repair | 132 |
| 3 | Notch1 | Notch signaling receptor; governs cell fate decisions, immune cell differentiation, and tissue homeostasis | 127 |
| 4 | Lef1 | Wnt/β-catenin pathway transcription factor; regulates cell proliferation and differentiation, and maintains cartilage homeostasis | 110 |
| 5 | Klf4 | Transcription factor; regulates cell cycle, phenotypic switching, and inflammatory responses | 63 |
| 6 | Hmox1 | Antioxidant enzyme; confers cytoprotection against oxidative stress and ferroptosis | 59 |
| 6 | Nt5e | Ecto-5'-nucleotidase; converts AMP to adenosine and mediates immunomodulation | 59 |
| 8 | Gli1 | Hedgehog pathway transcription factor; regulates stem/progenitor cell proliferation and tissue repair | 56 |
| 9 | Ccl3 | Pro-inflammatory chemokine; promotes immune cell recruitment and amplifies inflammatory cascades | 42 |
| 10 | Ngf | Neurotrophin; promotes neuronal growth and differentiation, and mediates pain signaling | 39 |

**
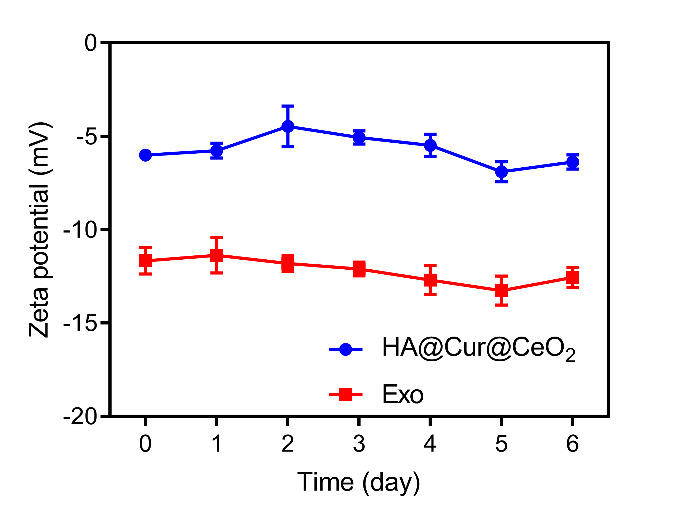
**

**Figure S1.** The stability of zeta potential within 6 days.


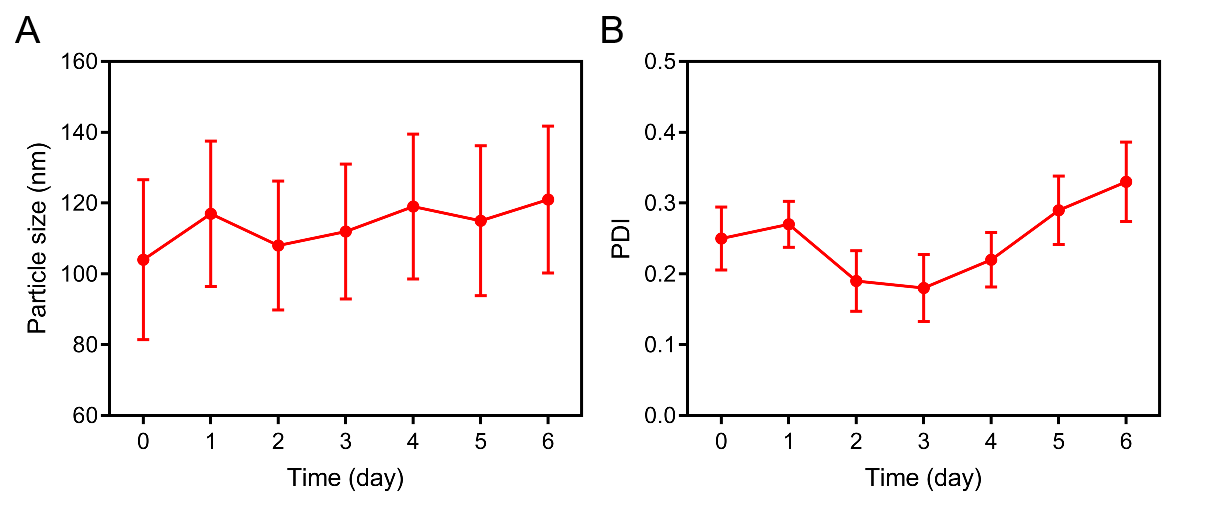


**Figure S2.** Hydrodynamic particle size (A) and PDI (B) of Exo within 6 days.

**
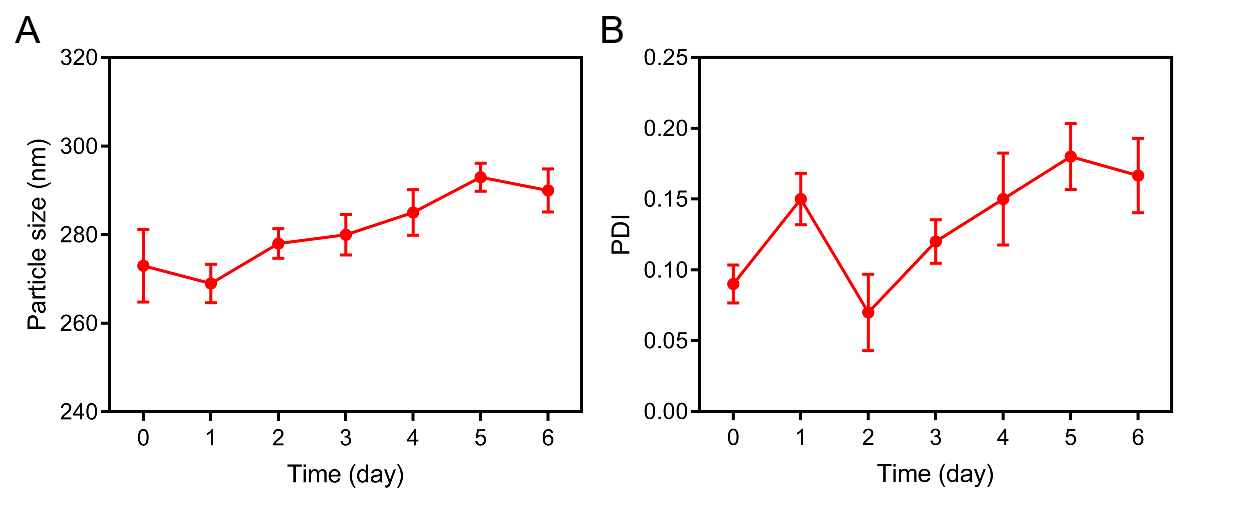
**

**Figure S3.** Hydrodynamic particle size (A) and PDI (B) of HA@Cur@CeO_2_ within 6 days.





**Fig. S4**. FTIR spectra of HA@Cur@CeO₂.


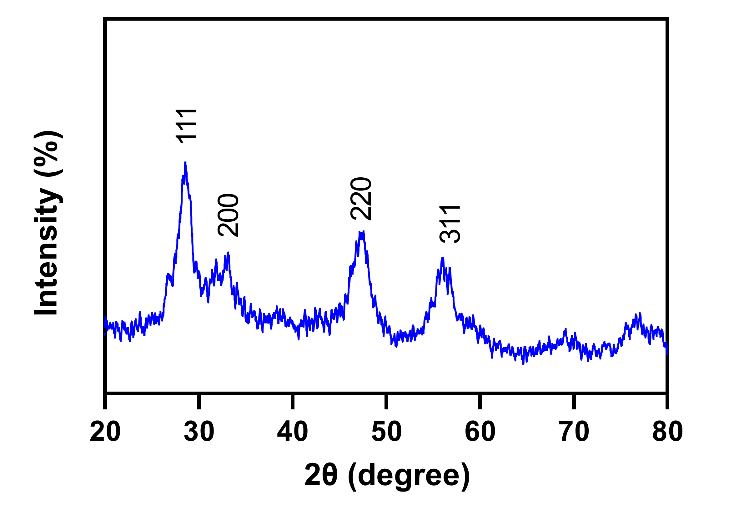


**Fig. S5.** XRD patterns of HA@Cur@CeO₂.


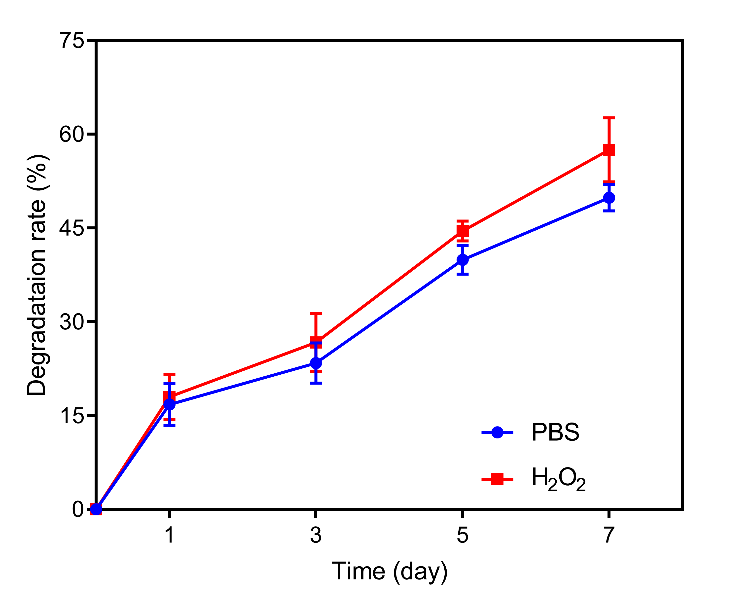


**Fig. S6.** Degradation profiles of the hydrogel under normal physiological and high-ROS conditions.


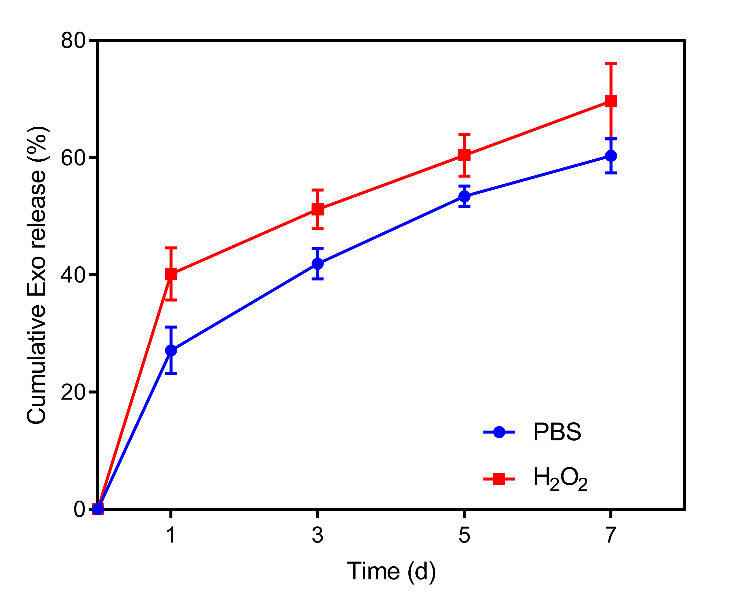


**Fig. S7.** The release curves of Exo from the hydrogel under normal physiological and and high-ROS conditions.


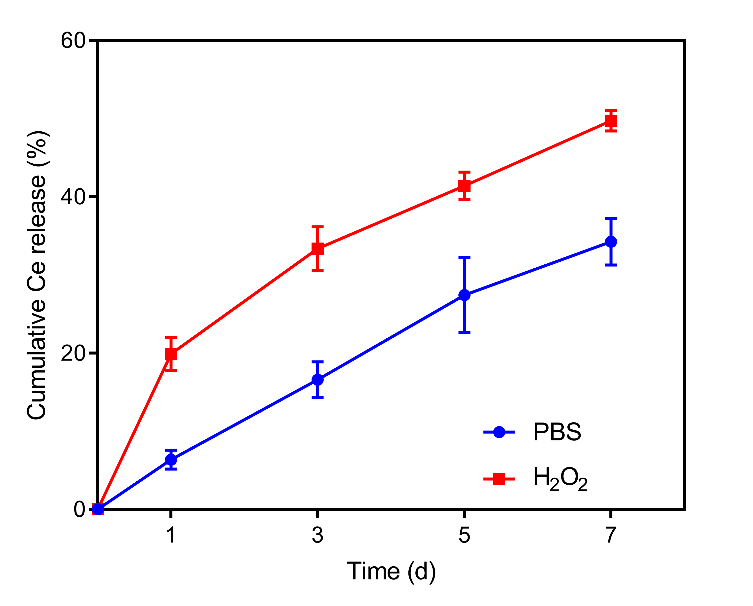


**Fig. S8.** The release curves of Ce from the hydrogel under normal physiological and and high-ROS conditions.

**
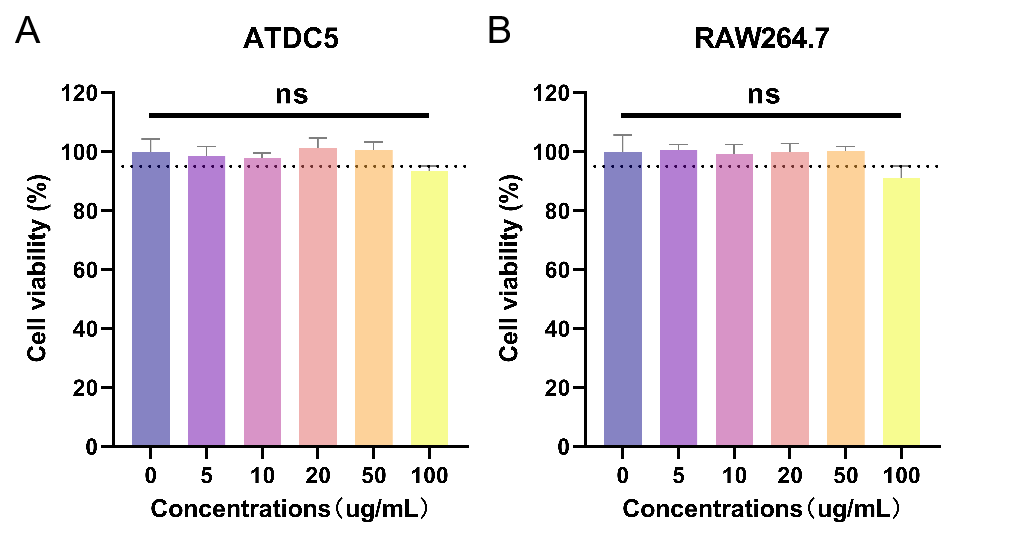
**

**Figure S9.** Cell viability of ATDC5 (A) and RAW264.7 (B) cells at different HA@Cur@CeO_2_ concentrations.

**
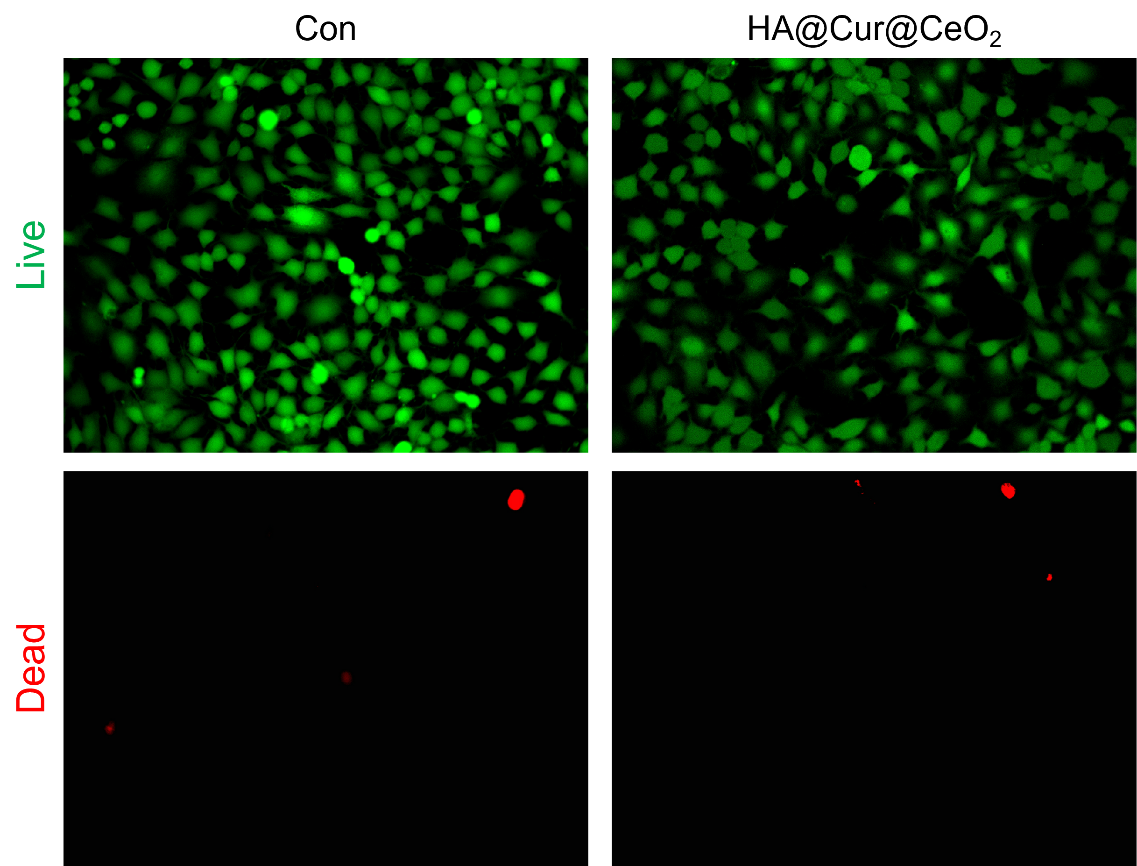
**

**Figure S10.** Live/dead staining of ATDC5 cells cultured with HA@Cur@CeO_2_.

**
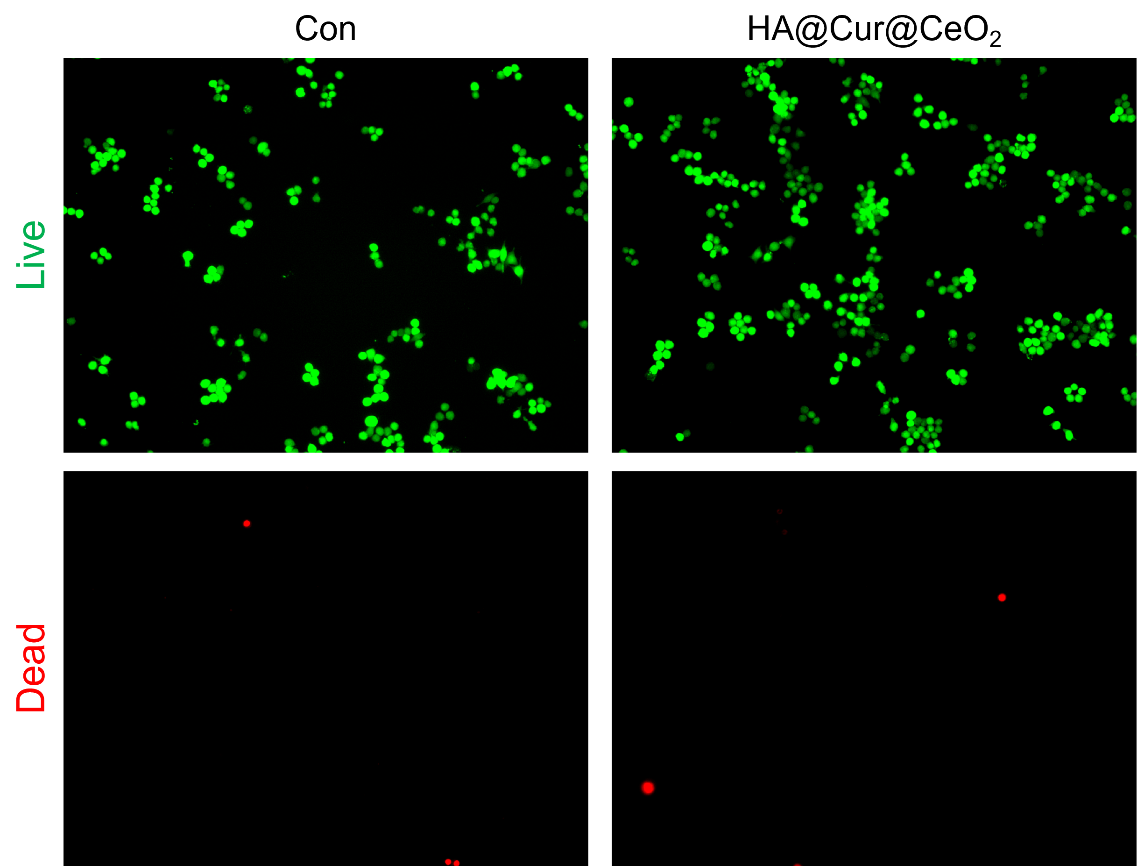
**

**Figure S11.** Live/dead staining of RAW264.7 cells cultured with HA@Cur@CeO_2_.


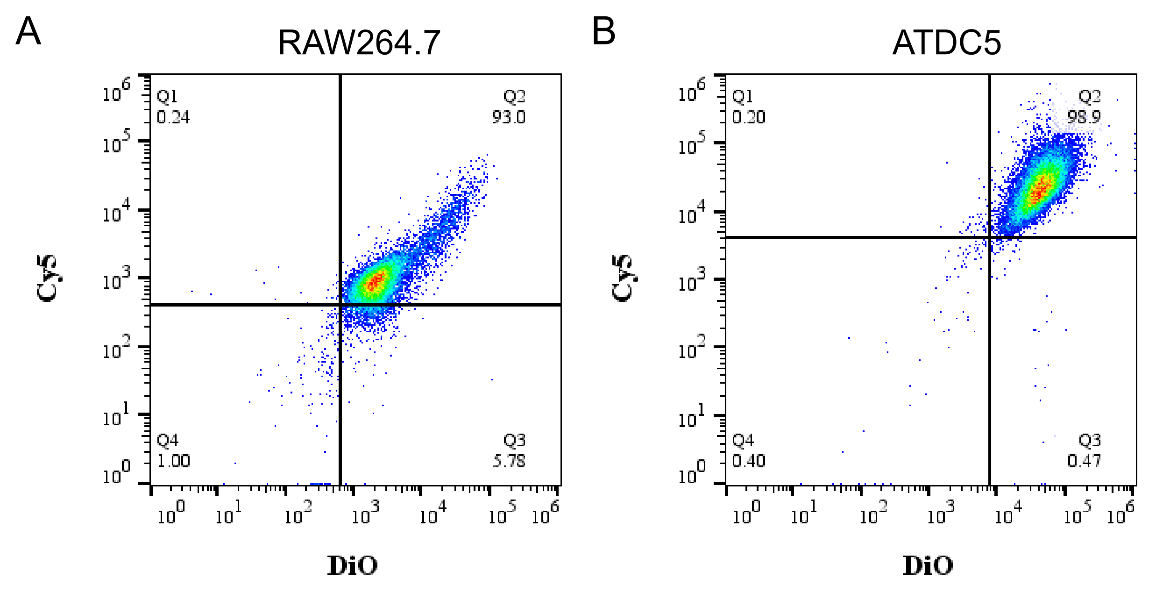


**Figure S12.** Flow cytometry for Exo and HA@Cur@CeO₂ uptake in RAW264.7 and ATDC5 cells.


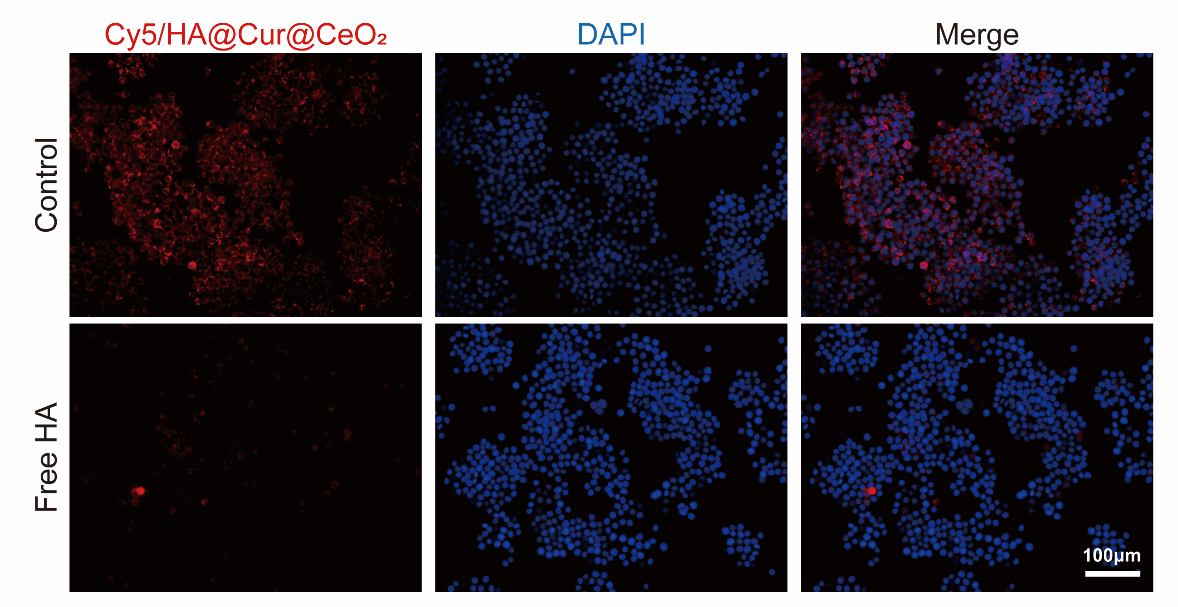


**Figure S13.** HA competition reduces the uptake of Cy5-labeled HA@Cur@CeO₂ in RAW264.7 cells.


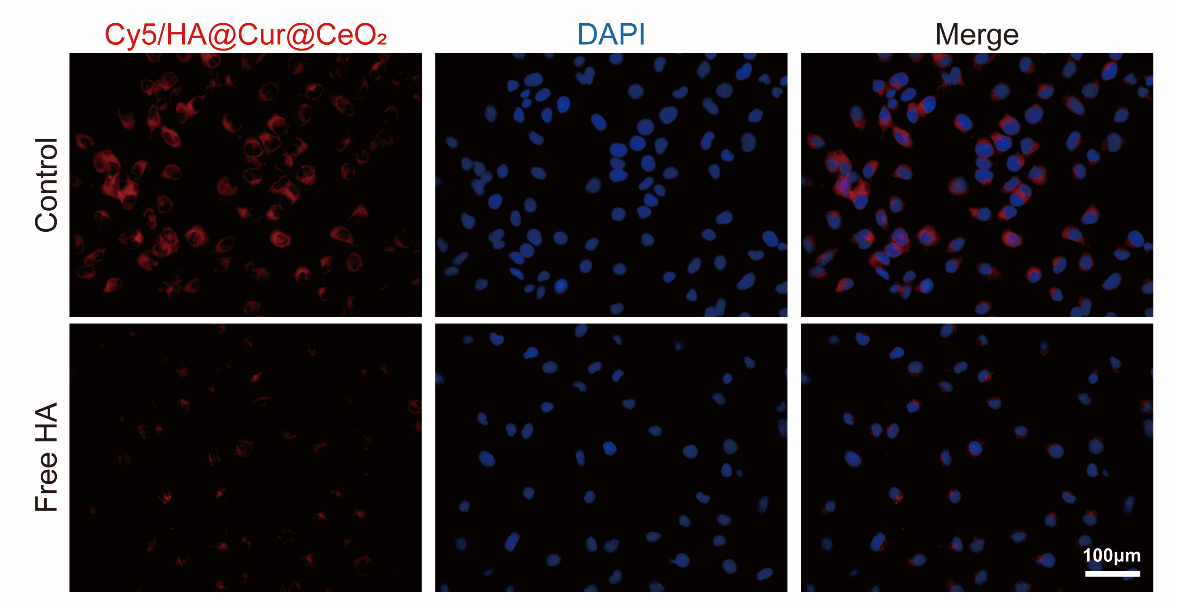


**Figure S14.** HA competition reduces the uptake of Cy5-labeled HA@Cur@CeO₂ in ATDC5 cells.

**
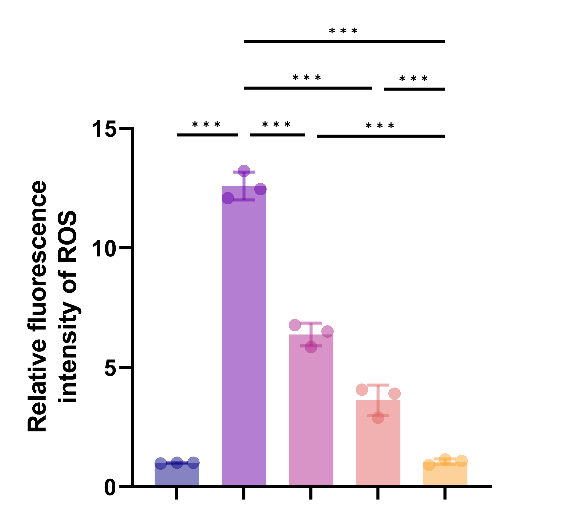
**

**Figure S15.** Relative fluorescence intensity of ROS in RAW264.7 cells.

**
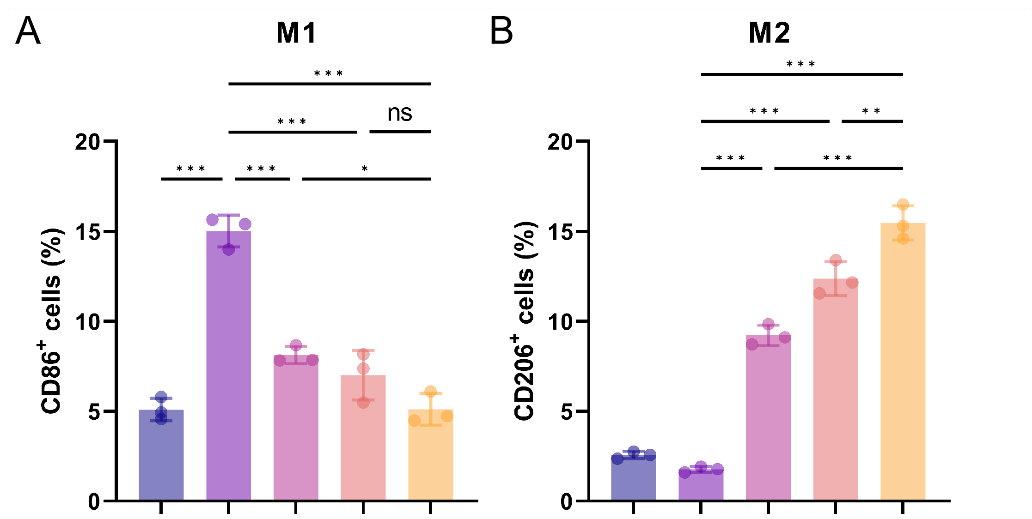
**

**Figure S16.** Proportion of M1 (A) and M2 (B) cells.

**
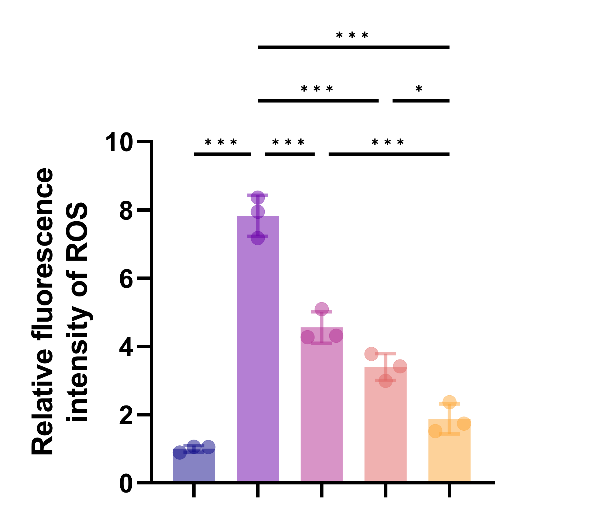
**

**Figure S17.** Relative fluorescence intensity of ROS in ATDC5 cells.

**
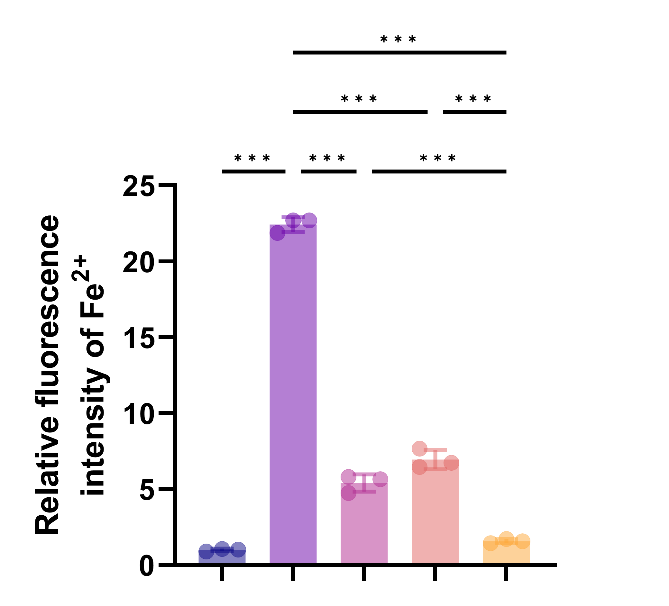
**

**Figure S18.** Relative fluorescence intensity of Fe^2+^ in ATDC5 cells.

**
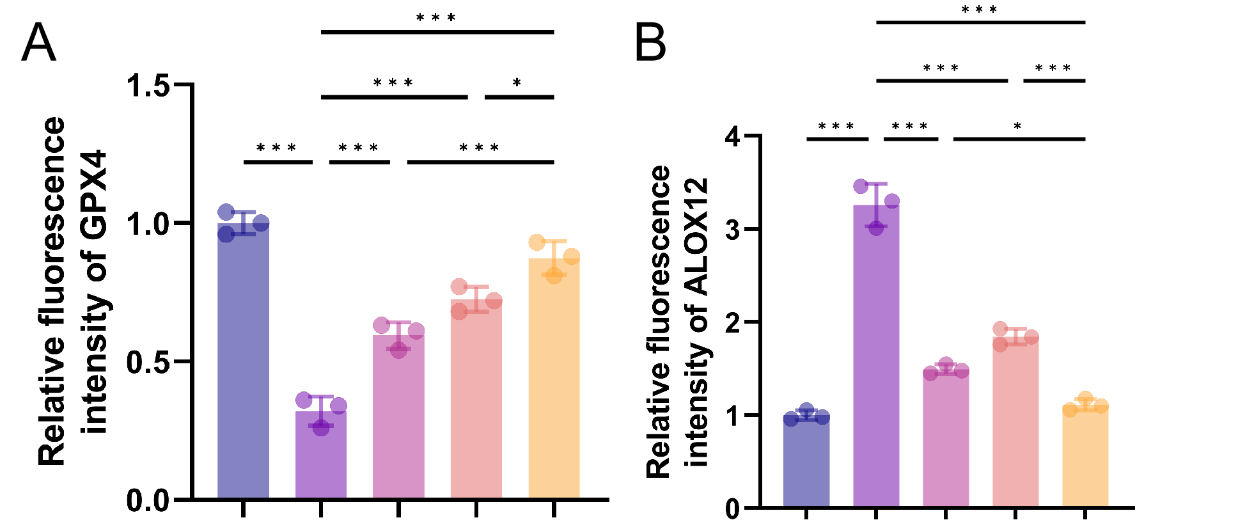
Figure S19.** Immunofluorescence semi-quantitative analysis of GPX4 and ALOX12 in ATDC5 cells.


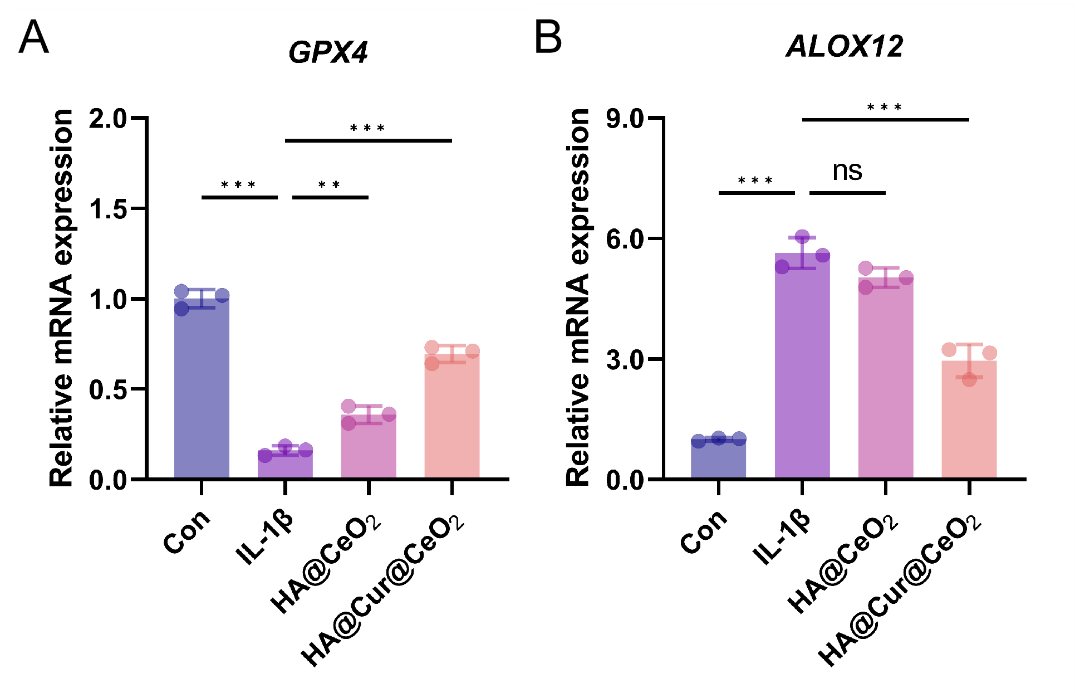


**Figure S20.** Comparison of the effects of HA@CeO₂ and HA@Cur@CeO₂ on GPX4 and ALOX12 in ATDC5 cells.

**
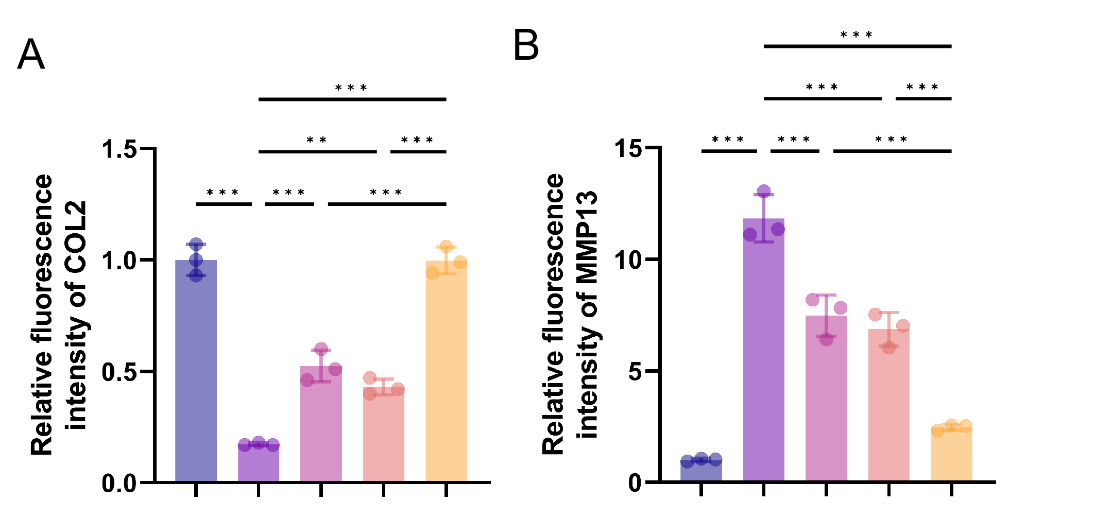
**

**Figure S21.** Immunofluorescence semi-quantitative analysis of COL2 and MMP13 in ATDC5 cells.

**
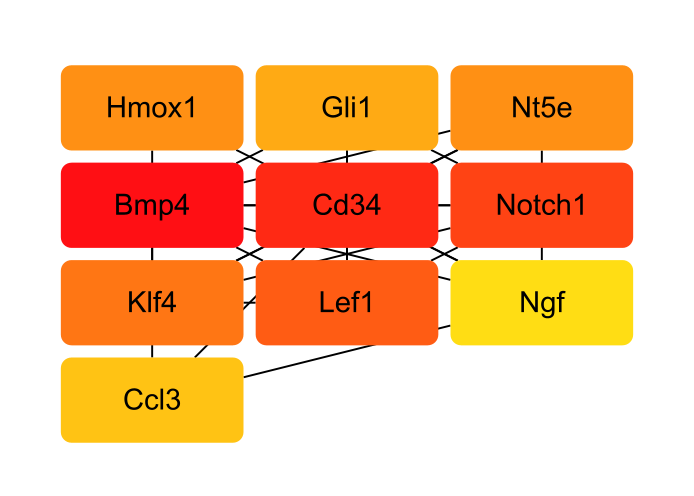
**

**Figure S22.** The hub genes with the top 10 degree.

**
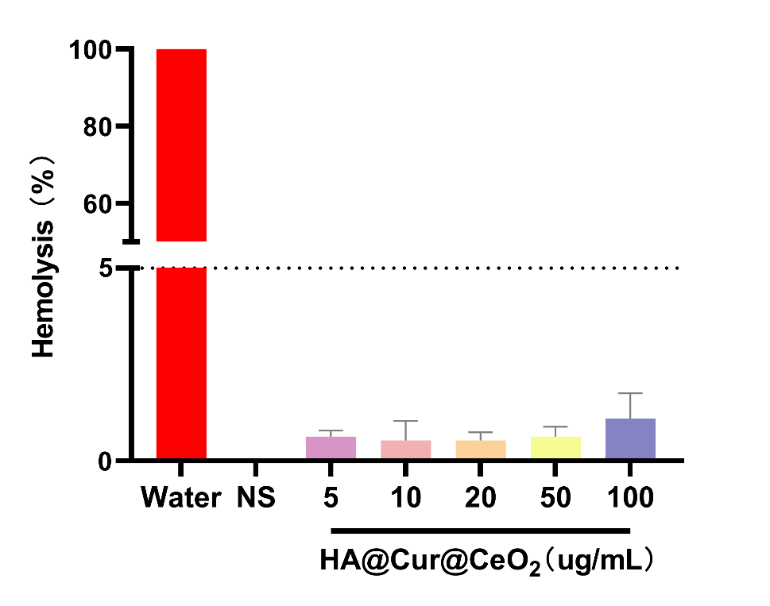
**

**Figure S23.** Hemolysis test with different concentration of HA@Cur@CeO_2_.

**
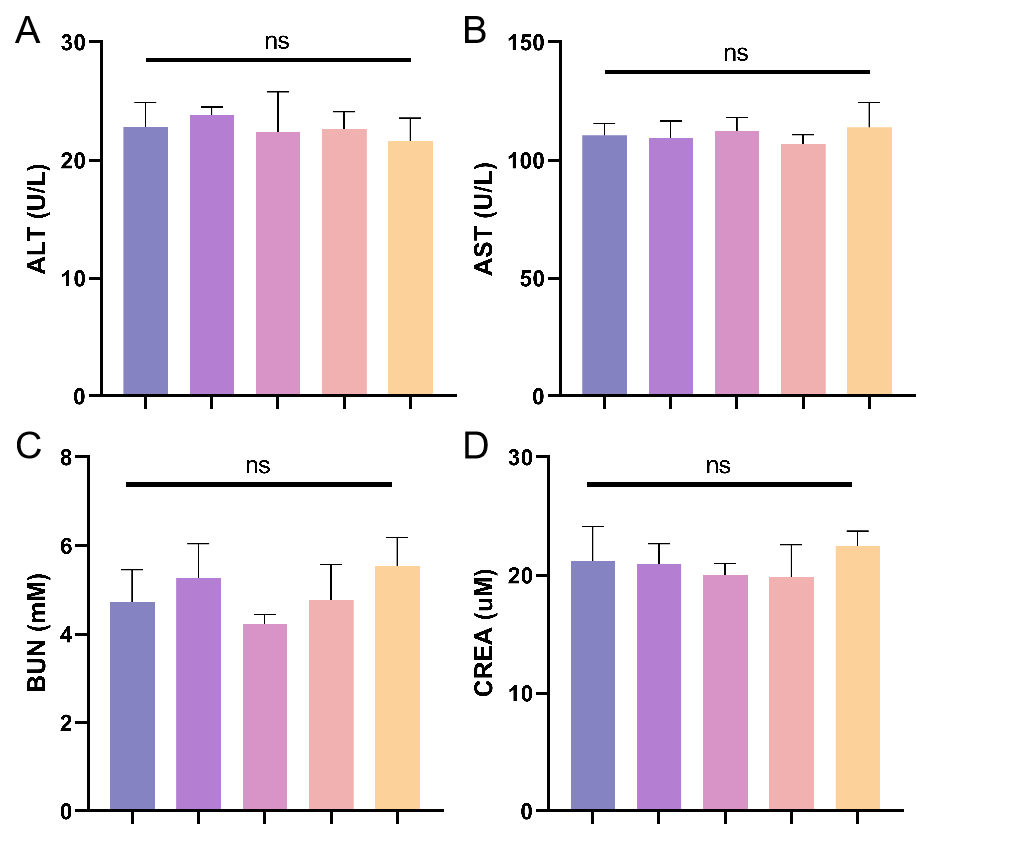
Figure S24.** Biochemical tests after different treatments.

**
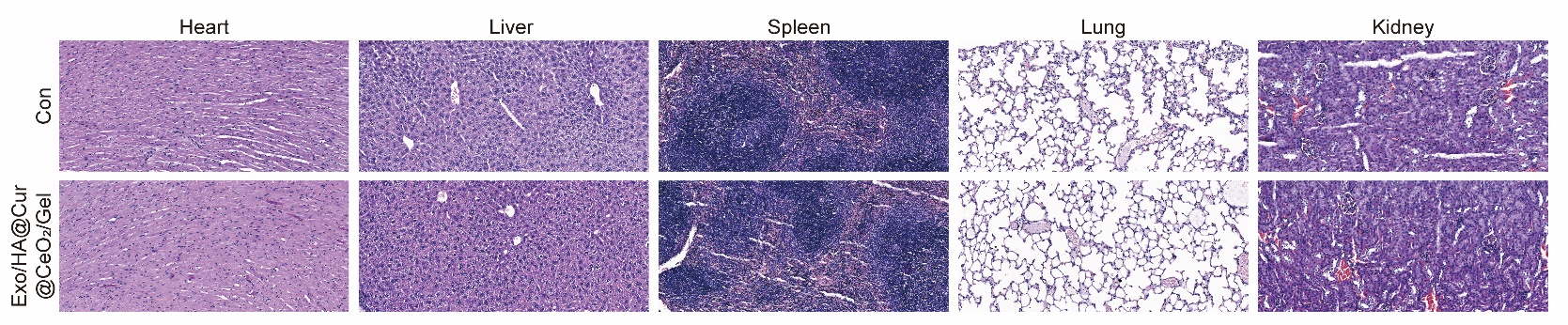
Figure S25.** HE staining of major organs (heart, liver, spleen, lung, and kidney) after Exo/HA@Cur@CeO_2_/Gel treatment.
